# Genomic surveillance of Canadian airport wastewater samples allows early detection of emerging SARS-CoV-2 lineages

**DOI:** 10.1038/s41598-024-76925-6

**Published:** 2024-11-03

**Authors:** Alyssa K. Overton, Jennifer J. Knapp, Opeyemi U. Lawal, Richard Gibson, Anastasia A. Fedynak, Adebowale I. Adebiyi, Brittany Maxwell, Lydia Cheng, Carina Bee, Asim Qasim, Kyle Atanas, Mark Payne, Rebecca Stuart, Manon D. Fleury, Natalie C. Knox, Delaney Nash, Yemurayi C. Hungwe, Samran R. Prasla, Hannifer Ho, Simininuoluwa O. Agboola, Su-Hyun Kwon, Shiv Naik, Valeria R. Parreira, Fozia Rizvi, Melinda J. Precious, Steven Thomas, Marcos Zambrano, Vixey Fang, Elaine Gilliland, Monali Varia, Maureen Horn, Chrystal Landgraff, Eric J. Arts, Lawrence Goodridge, Devan Becker, Trevor C. Charles

**Affiliations:** 1 University of Waterloo, Waterloo, ON, Canada; 2 University of Guelph, Guelph, ON, Canada; 3 Western University, London, ON, Canada; 4 Greater Toronto Airports Authority, Mississauga, ON, Canada; 5 Regional Municipality of Peel, Mississauga, ON, Canada; 6 Toronto Public Health, Toronto, ON, Canada; 7 Regional Municipality of York, Newmarket, ON, Canada; 8 Public Health Agency of Canada, Ottawa, ON, Canada; 9 Metagenom Bio Life Science Inc., Waterloo, ON, Canada; 10 Wilfrid Laurier University, Waterloo, ON, Canada

**Keywords:** Molecular biology, Next-generation sequencing, RNA sequencing

## Abstract

The severe acute respiratory syndrome coronavirus 2 (SARS-CoV-2) pandemic has shown wastewater (WW) surveillance to be an effective means of tracking the emergence of viral lineages which arrive by many routes of transmission including via transportation hubs. In the Canadian province of Ontario, numerous municipal wastewater treatment plants (WWTPs) participate in WW surveillance of infectious disease targets such as SARS-CoV-2 by qPCR and whole genome sequencing (WGS). The Greater Toronto Airports Authority (GTAA), operator of Toronto Pearson International Airport (Toronto Pearson), has been participating in WW surveillance since January 2022. As a major international airport in Canada and the largest national hub, this airport is an ideal location for tracking globally emerging SARS-CoV-2 variants of concern (VOCs). In this study, WW collected from Toronto Pearson’s two terminals and pooled aircraft sewage was processed for WGS using a tiled-amplicon approach targeting the SARS-CoV-2 virus genome. Data generated was analyzed to monitor trends of SARS-CoV-2 lineage frequencies. Initial detections of emerging lineages were compared between Toronto Pearson WW samples, municipal WW samples collected from the surrounding regions, and Ontario clinical data as published by Public Health Ontario. Results enabled the early detection of VOCs and individual mutations emerging in Ontario. On average, the emergence of novel lineages at the airport preceded clinical detections by 1–4 weeks, and up to 16 weeks in one case. This project illustrates the efficacy of WW surveillance at transitory transportation hubs and sets an example that could be applied to other viruses as part of a pandemic preparedness strategy and to provide monitoring on a mass scale.

**Supplementary Information:**

The online version contains supplementary material available at 10.1038/s41598-024-76925-6.

## Introduction

The severe acute respiratory syndrome coronavirus 2 (SARS-CoV-2) pandemic has had a global impact on individuals and economies. Cross-border international travelers, especially commercial airline travelers, facilitated the spread of SARS-CoV-2 to locations far from initial infection points, contributing to the rapid spread of SARS-CoV-2 worldwide^1–4^. While air-travel contributed to viral transmission across international borders, some evidence suggests that border closures enforced early in the pandemic did not reduce viral spread^5^.

Early in the SARS-CoV-2 pandemic, airport operations were critical to pandemic response for import/export of emergency personnel and medical supplies^6–8^. Toronto Pearson International Airport (Toronto Pearson) processed 40% of Canada’s air cargo prior to the pandemic and the average daily cargo activity more than doubled between 2019 and 2021^9^. The volume of cargo processed shows the importance of air cargo at Toronto Pearson locally, provincially, and nationally during the pandemic^9^. In addition to these more obvious functions during a pandemic, airports can also be an efficient and cost-effective point of wastewater (WW) surveillance for emerging or re-emerging pathogens arriving in a city, country, and region, while maintaining the anonymity of the travelers^2^.

Though WW surveillance is not a new technology for testing of infectious disease targets, the SARS-CoV-2 pandemic has provided an opportunity to modernize testing methods and target new pathogens, further illustrating the utility of WW as a means of community-wide epidemiological surveillance^10,11^. Sewershed sampling, including the influent of wastewater treatment plants (WWTPs), is a cost-effective and non-invasive strategy for capturing information on the presence and abundance of SARS-CoV-2 and the relative abundance of variants of concern (VOC) in defined communities serviced by different WWTPs^10,12–14^. WW samples can capture asymptomatic, pre-symptomatic, or mild cases that may not be captured in clinical surveillance data^12,15,16^. As clinical testing has been reduced, WW signals have provided a reliable and stable measure of community transmission^12^.

Detection of total SARS-CoV-2 in the waste of individual arriving aircraft or terminals using real-time quantitative PCR (RT-qPCR) has been used in many countries to track quantity of the virus in these transportation hubs^17–21^. Aircraft SARS-CoV-2 detection by RT-qPCR has been shown to be more sensitive than testing individual passengers prior to departure especially on long haul flights^18^. RT-qPCR detections from airport terminal WW can be predictive of local clinical case load^22,23^. When selecting WW sites for pathogen detection by sequencing to mitigate and/or track emerging outbreaks in a region or country, WW from airports should be considered for pandemic preparedness and outbreak management^24^.

SARS-CoV-2 evolution has resulted in a multitude of lineages and sub-lineages, some of which have increased transmissibility, immune evasion, or other advantages that make them of public health interest for surveillance^25,26^. Public Health Ontario (PHO) compiles and reports clinical whole genome sequencing (WGS) results to Public Health Units (PHUs) and GISAID which contributes to lineage definitions^27,28^. The data returned from sequencing of WW samples is comprised of sequence fragments of many individual viral genomes that must be disentangled from one another using bioinformatic tools, which use mutation frequencies to determine which lineages are present in each sample^29–31^. Individual mutation frequencies can be surveyed on their own, which is useful when the mutations in question contribute to immune evasion or are indicative of emerging VOCs. The spike protein mutation R346T is an example of such a mutation, which is present in many BQ* and XBB* lineages^32,33^.

WGS results from airport WW have been used to determine the introduction of variants into the surrounding communities^34–36^. WGS detections of Omicron sub-variants in airport WW samples from airports in Singapore and Amsterdam showed early detection compared to clinical as reported by the local public health agencies, and early detection compared to WWTPs as reported by a local environmental agency in the case of the Singapore study^23,36^.

As of March 2024, Toronto Pearson had direct flights to and from 61 countries which span North and South America, Africa, Asia and Europe^37^. Toronto Pearson has two terminals (Terminal 1 and Terminal 3) which both served domestic and international flights during the study period. Roughly a quarter of the number of weekly flights arrived from international destinations, while the other three quarters were split relatively evenly between destinations in the USA and elsewhere in Canada. Generally, airlines serving countries on the continents of Africa and South America were few and were being routed through Terminal 1 at the time of the study, while airlines serving countries on the continents of North America, Asia and Europe were more numerous, and were routed through both Terminals 1 and 3^37^.

Ontario WW surveillance was coordinated through the Ontario Wastewater Surveillance Initiative (WSI) at the time of this study. Site locations and program organization are explained in more detail in the methods section. The collaboration between academic labs, GTAA, and WSI PHUs has allowed research into the use of transportation hubs to monitor VOC arrival and emergence into Canada.

It was hypothesized that WW surveillance using WGS at a major international travel hub would provide early detections of variants of concern of SARS-CoV-2. In this study WGS results from WW surveillance at Toronto Pearson and the surrounding municipalities over a period of 14 months were generated and compared to illustrate the ability of WW surveillance at an international transport hub to provide early data on VOC introduction into the surrounding community. The observed early detections of SARS-CoV-2 lineages in airport WW compared to municipal WW and clinical WGS reporting provides an example of how sequence-based WW surveillance can be used to monitor viral threat emergence in communities via transportation hubs.

## Methods

### Site selection

**Fig. 1:**
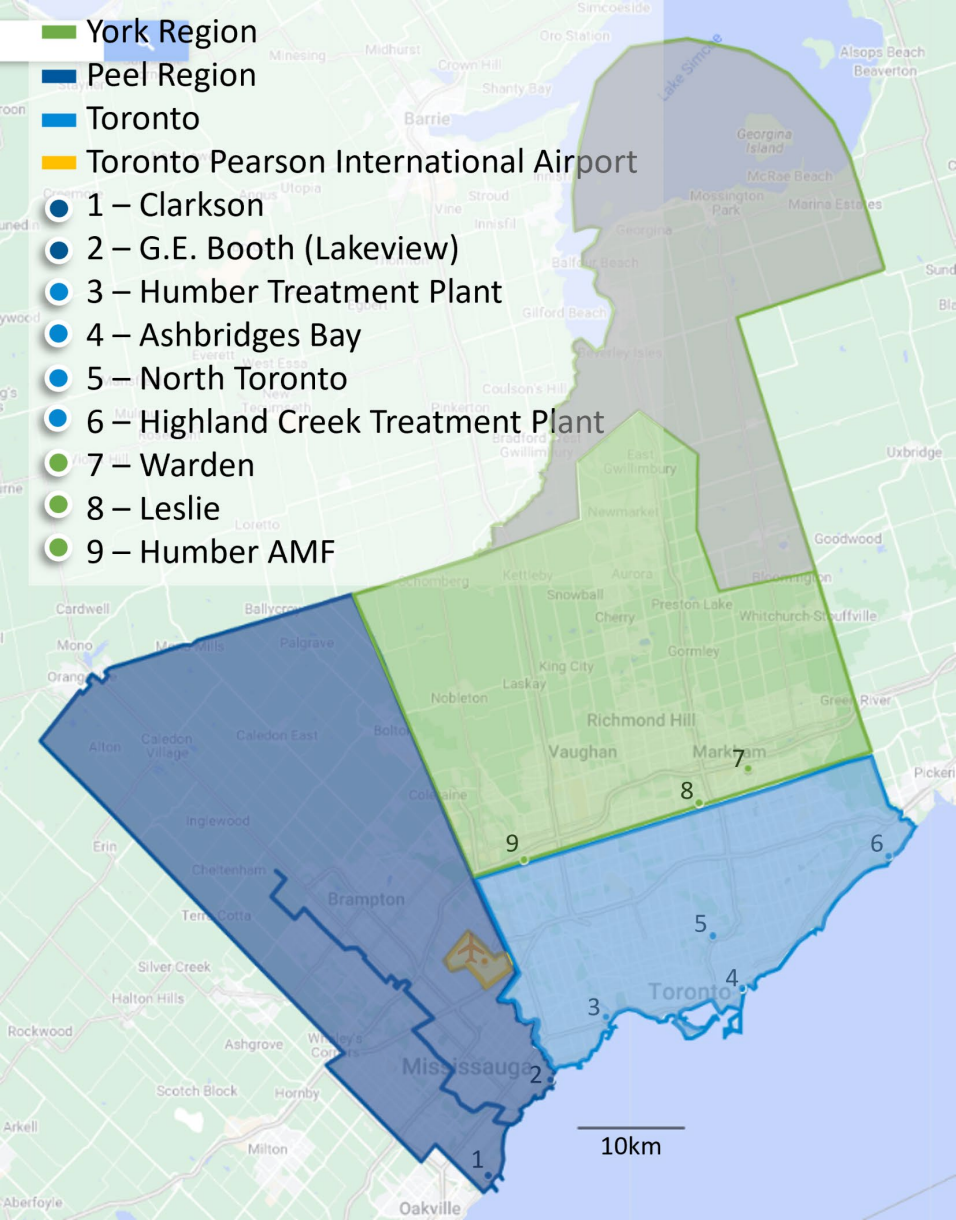
Map of sewersheds and sampling sites in the regions surrounding Pearson Airport.

The immediate area surrounding Toronto Pearson consists of three municipal regions, York, Peel, and Toronto, which include urban centers (Fig. 1). WW samples from these three regions were examined weekly, because these municipal regions were likely first contact points after the airport between passengers and the local population and would provide the best information about variant presence locally. Samples were received from two WWTPs in Peel Region (1 & 2), four WWTPs in Toronto (3, 4, 5, & 6), two sewage pumping stations in York Region (8 & 9) and one maintenance access facility (7). It should be noted that WW from Humber AMF (Site 9) flowed into Peel Region and was treated at the G.E. Booth (Lakeview) WWTP (Site 2). Site 7 included WW flow from Site 8 and flows collected from the north and northwest portions of York region.

Terminal sewers served departing and arriving passengers on flights routed through that terminal, as well as terminal staff, in the 24-h period preceding sample collection. Terminal samples were collected on-site from the sewers leading away from the building and did not contain WW from any other sources. Airplane lavatory sewage is emptied into trucks and carried to a triturator building at the airport, for disposal. Samples collected from this disposal point are referred to here as pooled aircraft sewage and contained sewage from most arriving aircraft in the 24-hour period preceding sample collection. Three samples per weekday were received from Toronto Pearson (pooled aircraft sewage, Terminal 1, and Terminal 3). WW from Toronto Pearson was then treated at WWTPs in both Peel and Toronto after joining the municipal WW sewer systems.

City of Toronto, Regional Municipality of York, and Regional Municipality of Peel, were all participants in the Ontario WSI coordinated and funded by the Ontario Ministry of the Environment, Conservation and Parks (MECP). In this program, each participating region collected municipal WW samples from WWTPs and pumping stations for analysis at academic labs and/or the PHAC National Microbiology Laboratory Branch (Winnipeg, MB). Toronto Pearson samples were collected by a third-party company and sent by courier to the academic labs for analysis, supported by Public Health Agency of Canada and coordinated by Ontario WSI.

### Sample collection

This study includes data from samples collected over the period of January 1st 2022 to March 31st 2023. WW samples from two airport terminals at Toronto Pearson (Terminal 1 and Terminal 3) were collected using passive torpedo samplers (soaked for 24 h each), until March 21st 2022 after which Terminal samples were collected using autosamplers that took one grab sample each half hour over a 24 h period to create a composite sample. Pooled aircraft sewage was also first collected from January 1st 2022 until October 21st 2022, using passive torpedo samplers. After October 21st 2022, pooled aircraft sewage was collected using an autosampler in the same way as the Terminal samples yielding 24 h composite samples. The switch to autosamplers was reliant on the installation of autosamplers at each location and the terminals were prioritized due to the WW flow at these sites damaging the passive samplers. Samples from municipal WWTPs were collected similarly by autosampler in the case of sites 1–6 and 9 or by grab sampling (single timepoint collection) in the case of sites 7 and 8, at pumping stations or pipes. Sample metadata including sample type, coverage statistics and sample preparation method is available in Supplementary Table S1.

### Library preparation and WGS

As part of the Ontario WSI, for each site included in this study, WGS was conducted by one of three partners at University of Guelph, University of Waterloo, and University of Western Ontario. The following section details which samples were processed by each lab, and how the procedures and reagents used differed.

WW samples were subjected to RT-qPCR to measure the quantity of SARS-CoV-2 present in the samples prior to sending them to one of the sequencing labs, or for the Terminal and Aircraft Sewage samples, the RT-qPCR was performed directly in the sequencing lab. All sequenced samples are summarized in Table 1. The SARS-CoV-2 concentrations in airport samples were stable throughout the sampling period (Ct scores between 29 and 31, Supplementary Table S1). During this study, municipal samples that were part of the WSI program with Ct scores above 35 were not sequenced, thus all municipal samples included here have Ct scores ≤ 35. Gene copy measurements for the entire Ontario WW qPCR dataset were published as copies/L values for all municipal samples included in this study^38^.

One sample per weekday (Monday - Friday excluding statutory holidays) was collected from Toronto Pearson Terminal 1 and Terminal 3 for sequencing at University of Waterloo. One sample per week was collected for sequencing at University of Waterloo from each of the following municipal WWTPs, pumping stations, or pipes: Leslie Street Pumping Station - York Sewershed (Site 7), Warden and 407 - York Sewershed (Site 8), Humber AMF Pumping Station - York Sewershed (Site 9), Clarkson South-Peel Water Pollution Control Plant (Site 1), and G. E. Booth (Lakeview) WWTP (Site 2).

In preparation for sequencing, the viral content of 20 mL of a WW sample was concentrated using Nanotrap Microbiome A Particles (Ceres Nanosciences) following the manufacturer’s protocol “Manual Nanotrap^®^ Wastewater Protocol using QIAGEN QIAamp^®^ Viral RNA Mini Kit”, section A. “Viral Capture” with the following modifications: whenever a magnetic stand was called for, the sample with beads was instead centrifuged at 8,000 × g at 4 °C for 4 min, and the pellet was resuspended in a small volume of remaining supernatant instead of water to transfer the sample to a smaller tube. In the case of Terminal 1 and 3 samples prior to March 21st 2022 one of 3 gauzes from the torpedo passive sampler was first shaken in 40 mL sterile PBS with 0.05% Tween 80 and antifoam reagent, wrung out, and the PBS with resuspended biosolids was used as the input material for the Nanotrap Microbiome A Particles (Ceres Nanosciences) concentration. The beads were resuspended in 700 µL of prepared Lysis Buffer (RLT + 2ME) for RNA extraction automated on a QIAcube Connect using a RNeasy mini kit (Qiagen ID: 74116). RNA was then reverse transcribed using LunaScript RT SuperMix Kit (NEB #E3010) and amplified using Q5^®^ High-Fidelity 2× Master Mix (NEB cat# M0492L) and the ARTIC V4.1 NCOV-2019 Panel of primers (IDT; 10008554, designed by the ARTIC network) resulting in ~ 400 bp amplicons. Amplicon libraries were prepared using the Illumina DNA Library Prep kit (20060059) with an additional 0.8× AMPure XP (Beckman Coulter) single sided bead cleanup prior to tagmentation. Paired-end (2 × 250 bp) sequencing of the libraries was performed using a MiSeq (Illumina).

One sample per day Monday-Friday (excluding statutory holidays) was collected from the triturator for sequencing at University of Guelph. Pooled aircraft sewage samples were prepared and sequenced as described previously^39^. Briefly, samples from passive samplers were preprocessed by submerging one of the embedded gauzes in 50 mL sterile PBS with 0.05% Tween 80 and antifoam reagent and stomached for 2 min. Viral particles in the filtrate from passive sampler and in the liquid samples from autosamplers were concentrated using Nanotrap Microbiome A particles (Ceres Nanosciences). RNA extraction was performed using the QIAamp Viral RNA extraction Mini kit (QIAGEN) carried out on the QIAcube (QIAGEN) instrument according to the manufacturer’s extraction method. Complementary DNA was generated using the SuperScript™ IV First-Strand Synthesis System (Thermo Fisher) and amplified using the same ARTIC V4.1 primers and protocols as above for the University of Waterloo. The amplicon libraries were prepared using the Nextera XT DNA library prep kit (Illumina). Paired end (2 × 150 bp) sequencing of the libraries was performed on the Illumina MiniSeq system. A maximum of 24 samples were loaded per run.

One sample per week was sequenced at the University of Western Ontario from each of the four Toronto WWTPs: Humber (Etobicoke) Water Pollution Control Plant (Site 3), Main (Toronto-Ashbridges Bay) Water Pollution Control Plant (Site 4), North Toronto Treatment Plant (Site 5), and Highland Creek (Scarborough) Water Pollution Control Plant (Site 6). Ceres beads were not used to concentrate the virus, instead a 30 mL aliquot of the composite influent WW sample was centrifuged at 4200 × g at 4 °C for 20 min. The supernatant was discarded, except for approximately 500 µl used to re-suspend the solids pellet. The re-suspended solids pellet was used for RNA extraction as described above for University of Waterloo. CDNA, PCR amplification and Library preparation was performed as described above for University of Guelph. Paired-end (2 × 300 bp) sequencing was performed using an Illumina MiSeq.

**Table 1:** Summary of samples.

Site Name	Region	# samples passing QC (Ct < 35, BOC10 > 20%)	# samples collected/week	Sample type
Pooled Aircraft	Airport	130	5	Passive Torpedo Sampler 24 h
		91		Autosampler 24 h
Terminal 1		44	5	Passive Torpedo Sampler 24 h
		233		Autosampler 24 h
Terminal 3		46	5	Passive Torpedo Sampler 24 h
		246		Autosampler 24 h
Leslie Street PS	York	55	1	Grab
Warden 407		53	1	Grab
Peel OCF/Humber AMF		62	1	Autosampler 24 h
Clarkson	Peel	60	1	Autosampler 24 h
G.E. Booth		61	1	Autosampler 24 h
Humber (Etobicoke)	Toronto	76	1	Autosampler 24 h
Ashbridges Bay		56	1	Autosampler 24 h
North Toronto TP		54	1	Autosampler 24 h
Highland Creek		79	1	Autosampler 24 h

Table summarizing all samples analyzed for this study. Site names and regions have been shortened. Region names will be used to define groups of samples as shown throughout results. Number of samples sequenced minus samples that were discarded because the breadth of coverage (BOC) at 10 reads deep or greater was less than 20% is counted in column “# samples passing QC (Ct < 35, BOC10 > 20%)”. Sample type refers to how the sewage was collected.

### Data processing and frequency prediction

Fastq files for all samples were pre-processed using cutadapt to remove adapter sequences, minimap2 to map reads to a SARS-CoV-2 reference genome (NC_045512.2 – Wuhan-Hu-1), and samtools to generate coverage and mutation frequency data at all sites along the genome^40–42^. Mapped genomes with breadth of coverage (BOC) > 20% at a minimum read depth of 10 were then processed using Alcov, a tool which uses unique and shared mutations to predict frequencies of SARS-CoV-2 lineages^31^. Shared mutations were used to predict presence of sets of lineages while unique mutations were used to differentiate between individual sub-lineages. Mutations were only considered if the read depth at that location was greater than 10. Constellations defining each lineage in Alcov were built to include all mutations differing from the reference genome which occur in ≥ 90% of the specific lineage sequences on covSPECTRUM^33^. Alcov was used to generate heatmaps and csv tables of predicted frequencies of lineages as well as mutation frequencies, from which the values presented in this article were extracted and plotted.

A benchmarking study has been published comparing Alcov to eight other lineage prediction tools designed for use with WW sequencing^43^. This study concluded that Alcov performed well compared to these tools including well-known tools such as Freyja^43^.

### Data Transformation

Predicted lineage frequencies output by Alcov are complex and affected by genome coverage. Greater coverage of unique mutations for lineages in the mixed samples improves the ability of Alcov to make calls with more detail in sublineage separation. When fewer unique mutations are available, Alcov uses shared mutations to predict a lineage or will output an A-or-B statement in which more than one lineage is assigned to a frequency of occurrence. When an A-or-B statement is given, any of these listed lineages could account for the mutations detected.

To give the frequency of each lineage analyzed in this study (BQ*, BA.2.75*, BF*, XBB*, XBB.1.5*, XBB.1.16*, and XBB.1.9*), A-or-B statements were summed together and assigned to the parent lineage if all their components were descendants of the lineage in question. For example, for the BQ* lineage calls, an Alcov call of “BQ.1 or BQ.1.1 or BQ.1.2” would have been added to the total BQ* frequency for that sample, but an Alcov call of “BA.5 or BA.5.3 or BE.1 or BQ.1” would not have been summed into the BQ* frequency lineage. The asterisk denotes the inclusion of descendant lineages, which do not overlap, apart from XBB* which includes all the lineages which make up XBB.1.5*, XBB.1.9* and XBB.1.16* as well as other XBB* lineages. This summation of lineages was done using Python with the code in the supplemental methods with information found in the alias_key.json file published on the pango-designation github^44,45^. Summed frequencies per sample for each of the lineages analyzed in this study are listed in supplementary Table S2. Plots were created using ggplot2 in R with the code included in the supplementary methods.

In the figures tables presented, Terminals 1 and 3 at Toronto Pearson were grouped together as Airport Terminal 1 and 3. Pooled Aircraft Sewage includes data from the passive torpedo samplers from January 1, 2022 to October 21, 2022, and the triturator building autosampler after this point. Clarkson South-Peel Water Pollution Control Plant and G. E. Booth (Lakeview) WWTP were grouped together as “Peel”. York Sewershed – Leslie Street PS, York Sewershed – Warden 407 and York- Peel OCF/Humber AMF were grouped together as “York”. Humber (Etobicoke) Water Pollution Control Plant, Main (Toronto – Ashbridges Bay) Water Pollution Control Plant, North Toronto Treatment Plant, and Highland Creek (Scarborough) Water Pollution Control Plant were grouped together as “Toronto”. Clinical data from Ontario was pulled from Public Health Ontario’s Weekly Epidemiological Summaries for SARS-CoV-2 genomic surveillance in Ontario^27^. Epi-weeks in this study began at epi-week 1 being the first week of the year with at least 4 days in January, which matches the reports that the clinical data was collected from.

**Fig. 2:**
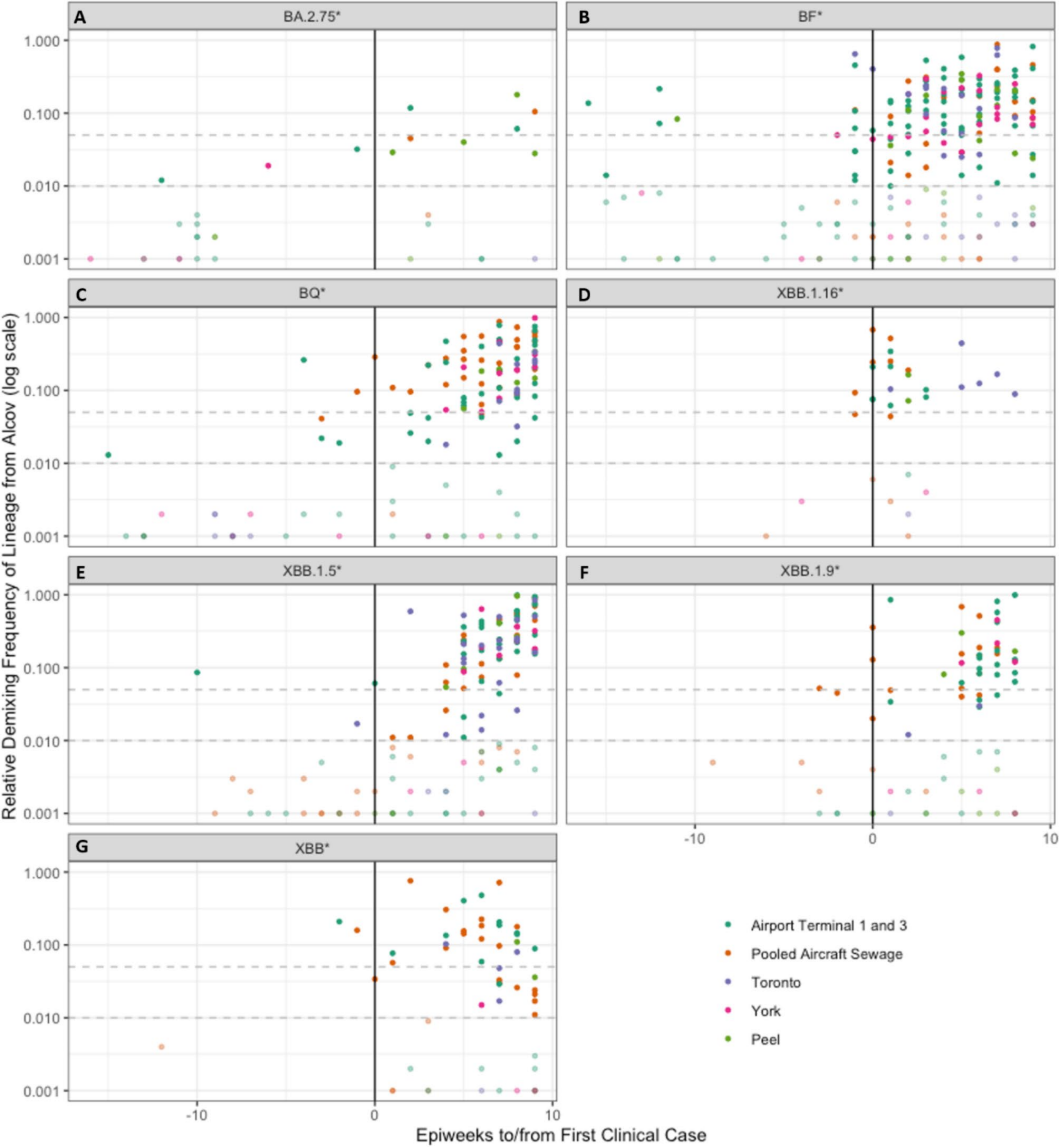
Frequency of lineages of interest from the de-mixing performed by Alcov (log sale). Horizontal dashed lines represent the 5% and 1% detection thresholds. Each point represents a single WW sample. Points with a frequency less than 1% are dimmed, as these are too low to consider a lineage as “detected”. The x-axis represents the weeks since the first PHO reported Ontario clinical case, with negative values representing detections in WW prior to clinical detections. Each panel represents a single lineage from the following list: BA.2.75* (**A**), BF* (**B**), BQ* (**C**), XBB.1.16* (**D**), XBB.1.5* (**E**), XBB.1.9* (**F**), XBB* (**G**).

**Fig. 3:**
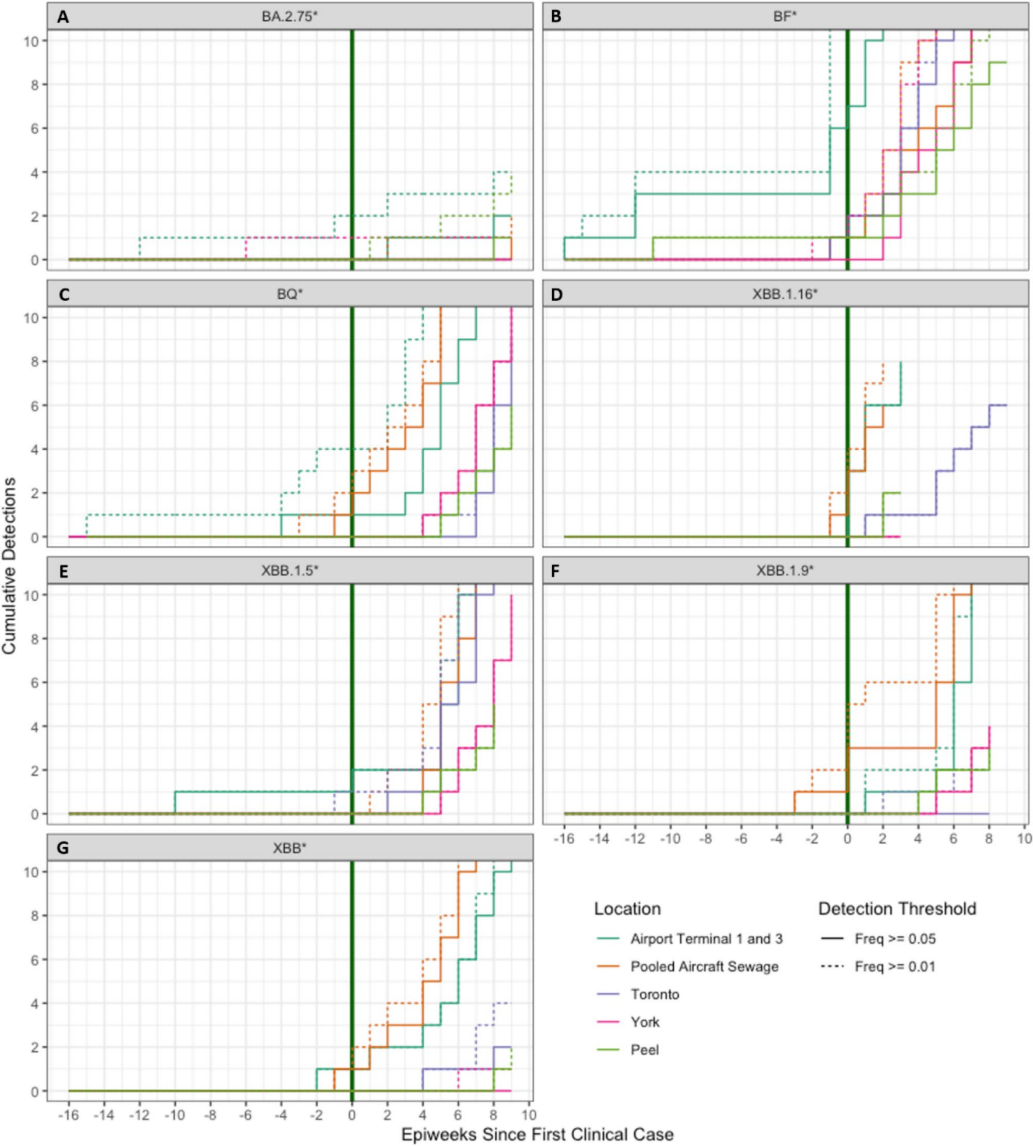
Cumulative detection probabilities for each lineage and location. Dotted lines represent detections with a 0.01 (1%) detection threshold, and solid lines represent detections with a threshold of 0.05 (5%). The x-axis represents the weeks since the first PHO reported Ontario clinical case, with negative values representing detections in WW prior to clinical detections. Each panel represents a single lineage from the following list: BA.2.75* (**A**), BF* (**B**), BQ* (**C**), XBB.1.16* (**D**), XBB.1.5* (**E**), XBB.1.9* (**F**), XBB* (**G**).

**Table 2:** Summary of first detections in WW and PHO report clinical data.

SARS-CoV-2 Lineage	Epi week of first detection in WW at Toronto Pearson and surrounding municipalities					Epi week of first clinical case (PHO)	Airport WW lead time	Municipal WW lead time
	Pooled Aircraft	Airport Terminals	Toronto municipal	Peel Region	York Region			
BQ*	2022–34	**2022–22 (33)**	2022–44	2022–42	2022–41	2022–37	15 weeks (4 weeks)	-
BA.2.75*	2022–31	**2022–17 (28)**	2022–42	2022–30	2022–23	2022–29	12 weeks (1 week)	6 weeks
BF*	2022–21	**2022–7 (10)**	2022–21	2022–12	2022–21	2022–23	16 weeks (13 weeks)	11 weeks
XBB*	**2022–39**	**2022–39**	2022–45	2022–48	2022–50	2022–41	2 weeks	-
XBB.1.5*	2023–1	**2022–39 (49)**	2022–47	2023–1	2023–1	2022–49	10 weeks (0 weeks)	2 weeks
XBB.1.16*	**2023–9**	2023–10	2023–11	2023–12	ND	2023–10	1 week	-
XBB.1.9*	**2023–2**	2023–6	2023–10	2023–9	2023–9	2023–5	3 weeks	-

Table summarizing first detection for each lineage in all 5 WW sample groups and PHO report clinical data. Detections were recorded as “year – epi week number”. Lead time was calculated from the first WW detection (bolded in each row) to the first clinical detection. If the lineage was not detected before March 31st, 2023, the corresponding cell reads “ND” instead of a date. In cases where the initial detection was more than two weeks ahead of any other detection in this study, the second detection was also considered in this table and the values were placed in brackets. A dash in the lead time column means that the WW detection occurred after the first clinical detection so there was no lead time.

**Table 3:** Summary of first detections in GISAID clinical submissions.

SARS-CoV-2 Lineage	Sample date of 1st detect -GISAID	Reported date - GISAID	Epi week of sample -GISAID	Epi week reported -GISAID	Accession #	Epi week of first detection in WW (Airport)	WW lead time
XBB.1.5*	2022-11-28	2022-12-12	48	50	EPI_ISL_16086975	39 (49)	9 weeks (– 1 week)
XBB.1.16*	2023-02-28	2023-04-24	9	17	EPI_ISL_17537606	9	0 weeks
BA.2.75*	2022-06-09	2022-06-21	23	25	EPI_ISL_13392500	17 (28)	6 weeks (– 5 weeks)
XBB*	2022-09-22	2022-10-03	38	40	EPI_ISL_15225593	39	– 1 week

Table showing first detection in WW only for comparison against first GISAID clinical sequence submission^28^.

**Table 4:** Summary statistics by location.

PHO reports comparison (weeks)				
Group	Detection threshold	Earliest first date	Mean first date	SD first date
Airport Terminal 1 and 3	0.01	16	– 4.5238095	6.845576
Airport Terminal 1 and 3	0.05	– 16	– 4.0996785	5.435883
Pooled Aircraft Sewage	0.01	– 3	0.1111111	3.027111
Pooled Aircraft Sewage	0.05	– 3	2.7529412	4.537951
Toronto	0.01	– 1	5.5263158	5.274345
Toronto	0.05	– 1	5.3373494	4.654599
York	0.01	– 6	5.0769231	7.398891
York	0.05	– 5	11.5607477	12.604415
Peel	0.01	– 11	3.4444444	5.972705
Peel	0.05	– 10	3.3375000	9.751266

GISAID comparison (days)				
Group	Detection threshold	Earliest first date	Mean first date	SD first date
Airport Terminal 1 and 3	0.01	– 59	– 1.181818	36.67102
Airport Terminal 1 and 3	0.05	– 59	3.590389	38.45018
Pooled Aircraft Sewage	0.01	– 6	21.142857	29.61511
Pooled Aircraft Sewage	0.05	– 3	55.414286	43.37450
Toronto	0.01	– 1	63.000000	46.18596
Toronto	0.05	– 1	57.378378	45.68136
York	0.01	– 3	67.500000	66.89224
York	0.05	4	114.903846	91.77870
Peel	0.01	20	57.538461	28.94712
Peel	0.05	46	119.384615	42.34794

Table showing summary statistics of first detections from all lineages across each location. Comparison to PHO reports is presented first in weeks, followed by comparison to GISAID in days. Statistics are calculated with detection thresholds of 0.01 (1%) followed by 0.05 (5%). The summaries were based only on the first detection of any lineage for each location. All dates refer to the number of weeks or days after the first clinical case, with negative values representing weeks or days before the first clinical case. The Mean first date is the mean of the first detection dates for the listed site that passed the Detection threshold listed in weeks or days after clinical detection of each lineage. The SD first date is the standard deviation of all first detection lead times. For GISAID comparison, only the 4 lineages which are searchable on GISAID were used for the calculations

## Results

### Coverage comparison by sample types

The dataset presented in this study includes sequencing data generated by different laboratories with slightly differing methods. The sampling methods at all sites were not kept consistent due to limitations of the sites themselves and the nature of some WW samples was inherently different, for example municipal WW and pooled aircraft sewage differed in concentration of solids compared to other sewage components, and the makeup of the other sewage components differed. The breadth of coverage for all samples in the dataset has been analyzed (Supplementary Table S3) to elucidate the difference in data quality as influenced by sequencing laboratory (with associated sequencing method), sample collection method, region, and sample type.

All groups of sequencing data analyzed had an average BOC greater than 65% and a median BOC greater than 75% of the reference genome. All regions had an average BOC between 65% and 81%. Pooled aircraft sewage had a median BOC of 96% and average BOC of 80.87%, which was higher than any other sample region including the terminals. Pooled aircraft sewage also had the highest standard deviation, likely due to the two different sampling methods producing different coverage results and the passive sampler data as a set having a high standard deviation. Terminals 1 and 3 together were most comparable to Peel region sites together (Median BOC 82% and 83.5% respectively), and York and Toronto regions had similar median BOC of 76%. Similarly, Guelph University’s samples had the highest median BOC (96%) followed by University of Waterloo (81%) and Western University (76%).

### Airport WW can provide an early warning of emerging VOCs

The raw sequencing data has been deposited to the Sequence Read Archive (SRA) under BioProject PRJNA1088471. Supplementary Table S2 shows processed lineage frequency results that were used to generate plots.

Emerging lineages and mutations of interest were tracked to compare first instances in airport WW, surrounding municipal WW, and clinical data where available. The lineages included in this study were BQ*, BA.2.75*, BF*, XBB*, XBB.1.5*, XBB.1.16* and XBB.1.9*. Only the sequencing results passing QC metrics as defined in the methods section were included in the following plots. A frequency of zero in these plots does not mean there was no SARS-CoV-2 detected in these samples, but rather that the specific lineage being queried was not found in the sample. Summed detection data for each of these lineages was plotted in Supplemental Figures S1 through S7 against epidemiological week (epi week), and the first detections were compiled and compared (Tables 2 and 3). Summed detection data was plotted in Fig. 2 against an X-axis normalizing all timelines of lineage emergence in terms of the first clinical detection as reported by PHO so that the WW detections could be visualized by lead time^27,46–51^.

When all BQ* lineage predictions were compiled for all the sites (Fig. 2C, Figure S1) results showed that the first detections occurred in the airport terminals in epi weeks 22 and 33, 15 and 4 weeks prior to PHO reported clinical detection, though the first detection was at a frequency less than 5%. Then at epi week 34 the pooled aircraft sewage had a small signal which continued to be the main detection until the Ontario clinical data first reported BQ* in epi week 37^47^. The surrounding municipal sites did not have a detection of BQ* until a small detection in epi week 40 (Toronto) and a slightly higher detection in epi week 41 (York region), both after the first PHO clinical sequence was collected.

BA.2.75* emerged in the Ontario clinical data in epi week 29 (Fig. 2A, Figure S2)^46^. Airport terminal BA.2.75* detections in WW occurred in epi weeks 17 and 28, 12 weeks and 1 week prior to PHO reported clinical detection, and the first detection in surrounding municipal samples was in York in epi week 23. All of these detections prior to the clinical first detections were at frequencies less than 5%. During post-processing of the data with Alcov there were some low frequency detections of BA.2.75 as early as February 14th 2022 in York region municipal WW and Terminal WW samples but these were not likely to be true detections. The coverage of samples with low frequency BA.2.75 detections in February and March 2022 were all below 60%, and these samples were predicted to contain parent lineage BA.2 when they were initially reported to PHUs and Toronto Pearson. BA.2.75 was not designated as a Pango lineage until June 2022, with detections earlier than June 2nd 2022 only reported from India^52^. WGS from India early in the pandemic was infrequently submitted and often had ambiguous sequence calls, causing scientists to recommend excluding early sequences from India when analyzing GISAID WGS data^53,54^. It is more likely that the low frequency detections that occurred in our dataset were another closely related BA.2* lineage so we have analyzed only detections which occur within 16 weeks of the first clinical detection by PHO through the end of the study period.

BF* lineages were detected in the airport terminals in epi weeks 7 and 10, 16 and 13 weeks ahead of PHO clinical detection respectively, and then in Peel (epi week 12), York (epi-week 21), in Toronto in epi week 21 and multiple times in the airport terminals between epi weeks 21 and 24 before Ontario’s first clinical case was reported in epi week 23 (Fig. 2B, Figure S3)^27^.

When XBB* lineages were analyzed as a group, detection occurred in Terminal and pooled aircraft WW during epi week 39, two weeks prior to the PHO initial clinical detection, and then in municipal WW first in Toronto in epi week 45, though consistent detection did not begin in municipal WW until epi week 47. The first clinical detection in Ontario took place during epi week 41 (Fig. 2G, Figure S4)^47^.

In addition to analysis of the XBB* lineages as a group, three specific sub-lineages of XBB were investigated: XBB.1.5*, XBB.1.16*, and XBB.1.9* as these seemed to be the most persistent. The first detection of an XBB.1.5* sub-lineage was in an airport terminal sample in epi week 39, 10 weeks prior to PHO initial clinical detection, and 8 weeks before the first municipal detection in a Toronto sample in epi week 47. There was another detection of XBB.1.5* in epi week 49, and the first clinical detection, also in epi week 49 ^49^. Consistent detection of XBB.1.5* in municipal and airport WW samples was not observed until epi week 1 of 2023 (Fig. 2E, Figure S5).

The first detections of XBB.1.16* were in pooled aircraft sewage during epi week 9, followed by detection in the airport terminals and the first clinical detection in epi week 10^50^, . The first detection in municipal WW occurred in epi week 11 in Toronto (Fig. 2D, Figure S6).

For XBB.1.9*, the first detections were all in the pooled aircraft sewage samples from epi weeks 2–6. The first clinical detection in Ontario did not take place until epi week 5, 3 weeks after the first airport WW detection, whereas the first detection in municipal WW was not until epi week 9 in Peel Region (Fig. 2F, Figure S7)^51^.

Cumulative detections above 1% and 5% over time per WW site were plotted in Fig. 3 to visualize the signal consistency after detection of each lineage (Fig. 3A-G). A stepwise increase in the cumulative detections each time a sample was taken is expected if a lineage was introduced into a community and remained present, either by spreading to other individuals or continuing to be shed by the original individuals, as each sample would have a detection of that lineage. More than one sample was collected each week for all sample groups represented in Fig. 3 so consistent detection appears as a line which increases on the y-axis (detections) faster than on the x-axis (weeks). Examples of consistent detection after initial detection were seen in the case of Toronto (purple) BF* (Fig. 3B) and York (pink) BF*, BQ* and XBB.1.5* (Fig. 3B, C and E). In most cases airport terminals 1 and 3 (teal) and pooled aircraft sewage (orange) detections plateaued after initial detection which means the initial detections were isolated instances and were not followed by consistent detection of the same lineage in the following weeks. After the clinical detection the airport WW sites showed consistent detection of the lineages.

GISAID EpiCoV™ is a searchable database for SARS-CoV-2 sequences that is accessible only to registered users. It was possible in June 2024 to filter the Ontario clinical sequences in this database by a sub-set of the lineages of interest focused on in this study, and the found first detections are summarized in Table 3^28^. PHO-submitted clinical data on GISAID included detections prior to those published in the risk assessment reports. When compared to the GISAID data that was available, Airport WW did not provide as much lead time.

Clinical first detections from PHO risk assessment reports were used as the primary dataset for comparison but clinical first detections from GISAID were also considered if available^28^. Sequence submissions which were available at the time of publication as representatives of a lineage on GISAID may not have been classified as that lineage during the study period when these lineages were emerging.

The country in which a lineage of interest originated could inform the possible routes of transmission before arrival in Ontario. Countries of origin were collected from the pango-designation GitHub and are as follows: BQ.1 originated in Nigeria, BA.2.75 was initially mainly found in India, BF.1 was initially found in England, Denmark, Spain and Scotland, XBB was initially found in USA and Singapore, XBB.1.5 originated in the USA, XBB.1.16 was initially found in India, USA, Singapore, and Europe, and XBB.1.9.1 was initially found in Indonesia, Singapore, Malaysia and England^44,45^. XBB*, XBB.1.5* and XBB.1.16* were associated with or originated in the USA, while other lineages were associated with or originated from countries on continents other than North America.

In Table 4 first detections were summarized statistically. Values were summarized by location, across all lineages. Frequencies greater than 1% or 5% were each used as thresholds to consider a lineage detected in the analysis. The airport terminals had the earliest average first detection lead time, with airplane sewage having the second earliest average lead time, but with lower variance and thus aircraft sewage had more consistent detections.

The lead time of each site compared to the PHO first detect or GISAID first detect was summarized statistically in Table 4, considering only the lineages which were searchable on GISAID for that portion of the table (Table 3)^28^. The values in this table are represented in days for the GISAID comparisons and weeks for the PHO report comparisons. While there were still some instances where WW detections preceded the GISAID detections which can be seen in the earliest first detection column, the mean first detections were for the most part positive numbers meaning that the WW detections usually occurred after the first sample on GISAID for a lineage.

### Mutations of interest provide an early warning signal for lineage groups

**Fig. 4:**
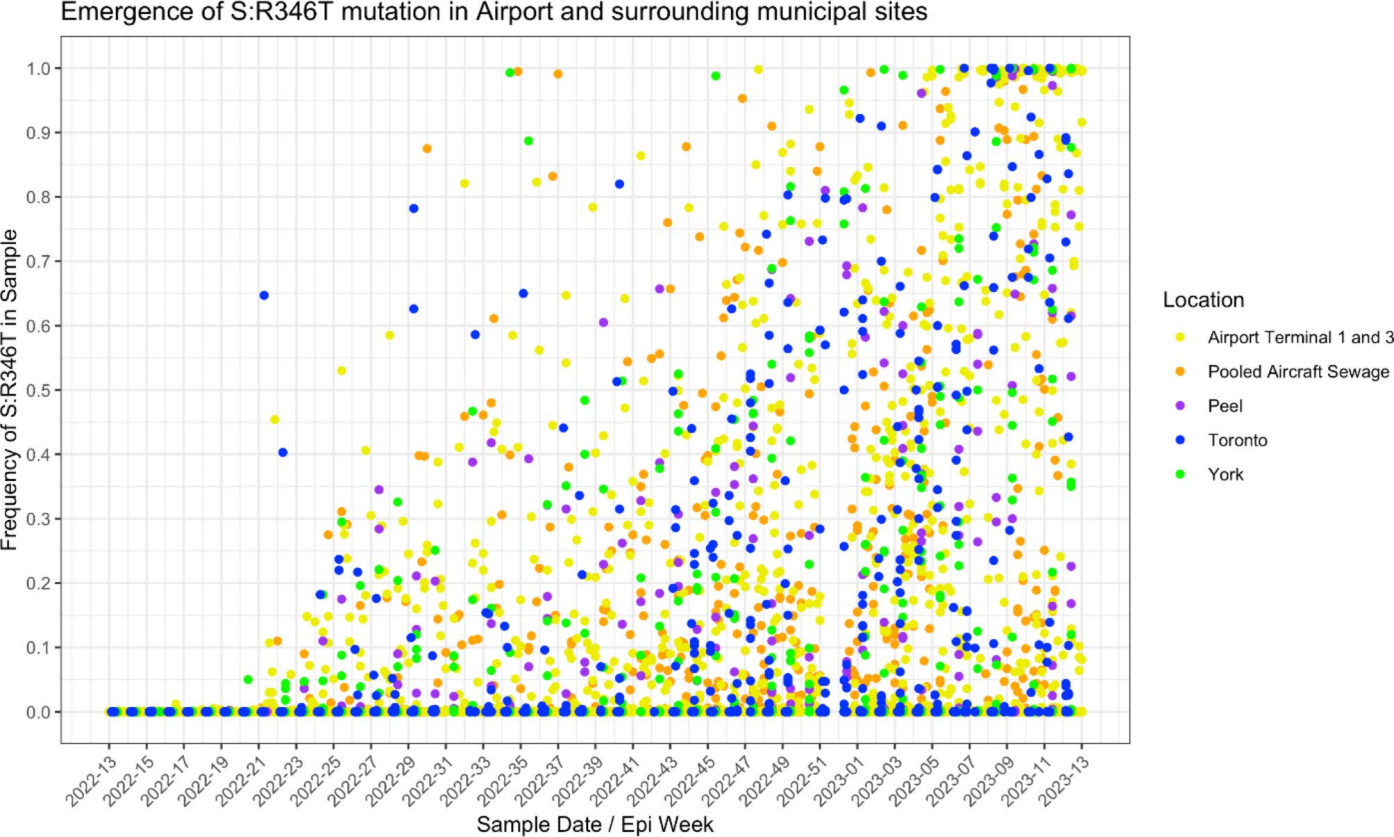
Frequency of the alert mutation S: R346T in WW samples from Toronto Pearson and surrounding municipal sites in Toronto, York, and Peel regions. Each point represents a single WW sample.

Over the pandemic, there were periods where it was increasingly difficult to track specific SARS-CoV-2 lineages in WW due to convergent evolution of mutations in the spike protein’s receptor binding domain. During this time, some WW surveillance programs followed certain important mutations. These mutations were deemed important for a variety of reasons, but the mutation examined here (S: R346T) is a mutation that aids SARS-CoV-2 lineages to escape antibodies, including the monoclonal antibodies used for treatment of COVID-19^32^. The S: R346T mutation was present in several lineages of SARS-CoV-2 that were circulating during the period of this study. The first instance of this mutation above 1% was a detection in a York Region sample in epi week 20, followed by several higher detections in terminals 1 and 3, pooled aircraft sewage and samples from Toronto region in epi weeks 21 and 22 (Fig. 4). There is no information about the first clinical detection of this mutation in Ontario.

## Discussion

Toronto Pearson is the largest international airport in Canada, hosting 40 airlines that fly to more than 155 cities worldwide^9^. In 2019, 50.5 million passengers travelled through the airport. There are nearly 50,000 people employed at the airport^55^. The diversity and number of people served by Toronto Pearson made it an extremely valuable site to monitor as part of joint efforts between Public Health Agency of Canada and the Ontario WSI program.

Sampling at Toronto Pearson through the WSI began in January 2022 making them the first Canadian international airport to serve as a regularly sampled WW surveillance site. To our knowledge, this is the first study to track lineages in Canadian airport terminal WW.

In this study there were several examples of initial detections of SARS-CoV-2 lineages from WW samples collected from Toronto Pearson (Terminals or Pooled Aircraft Sewage) ahead of a municipal WW detection with a lead time ranging from one week (BA.2.75*, XBB*, XBB.1.16*) to 4 weeks (BQ*) typically and up to 16 weeks (BF* lineages) before a clinical sample was detected in Ontario. A one-week lead time was more common. The average lead time for WW detections in Terminals 1 and 3 was 4 weeks at a detection threshold of 5% while aircraft sewage did not on average provide a lead time in this study (Table 4). The lineages and mutations followed in this study were chosen based on interest during the sampling program as identified by PHAC, Ontario Public Health Units, the Greater Toronto Airports Authority (GTAA), or by media coverage. The examples of early detection of emerging VOCs in Southern Ontario suggest that airport WW surveillance is a valuable tool for public health in pandemic preparedness as it can track populations at borders which can allow lead time to implement public health measures, such as masking and vaccination programs.

### Clinical sequences available on GISAID represent earlier cases of emerging variants

Throughout the study the clinical first detections as found in PHO reports were primarily used to measure lead time. The GISAID clinical sequences submitted by PHO may not reflect data availability at the time that these lineages were emerging as the data may have been transformed or re-analyzed since that time, while the PHO reports were accessed as they are referenced. It is also unlikely that end users of this data would search for emerging lineages on their own using the GISAID database. A study on variant detection by WGS in airport WW performed in Singapore reported even longer lead times for Omicron sub-variants and similarly used only local public health records for comparison^23^. For these reasons, the usefulness of WW surveillance at transportation hubs for informing public health of emerging lineages is not decreased by the shorter (or lack of) lead times when compared to GISAID data.

The presence of earlier GISAID clinical datapoints for the lineages BA.2.75*, XBB*, XBB.1.9* and XBB.1.16* indicated that the lineages were present in low abundance in Ontario prior to detection at Toronto Pearson (Tables 3 and 4). In cases where the countries of origin included the USA, it is likely that cross-border transmission occurred at land border crossings as often as or more frequently than through the international airport, which would provide a possible explanation. BA.2.75* was an interesting case however with only one very low frequency detection occurring before the GISAID detection, because it originated in India, so had potential to arrive in Ontario from overseas carried by airline travelers.

### Transient nature of airport WW and accuracy of first detections

Some considerations should be made for the transient populations contributing to the airport sampling sites. Terminal samples provided information on both the transient population of travelers (people who may have been terminating their journey in Southern Ontario or connecting onward to other destinations) and the more consistent population of airport staff and associates who worked in close proximity to the travelers. Pooled aircraft sewage on the other hand mostly represented the transient populations made up of travelers and mobile aircraft staff. For both airport sampling sites, multiple signal spikes occurred which dropped again before a new lineage gained a foothold in Ontario and more consistent detection of a lineage in municipal WW occurred. Each spike could represent a single case or a few cases passing through the airport, and there is no way to know the ultimate destination of the person or persons carrying the virus. The result is that each initial detection, even if isolated, should be treated as valuable data if it is going to serve as an early warning signal for in-country transmission.

The analysis of cumulative detections per site per lineage (Fig. 3) show that in most instances the time between initial and subsequent or consistent detection in Terminal 1 and 3 WW was longer compared to both the pooled aircraft sewage and municipal WW regions. This was expected when comparing municipal and terminal WW because transmission is less likely to occur within the population as the infected individuals may have been in transit. If transmission did occur within a terminal, the secondarily infected individuals may not have contributed to the sampled WW before departing. It was expected that the pooled aircraft WW would show the same lag between initial detection and subsequent detections given the transient nature of the population represented but pooled aircraft sewage did not lag to the same degree. It is possible that the passive samplers used initially were less sensitive, causing the detections that were observed after the switch to autosamplers to represent higher frequency lineage presence.

It is not typical to place confidence in single detections from WW surveillance due to the nature of the sample type, being comprised of many different individual viral particles and with each particle contributing a partially degraded genome. Frequency prediction tools are necessary to ‘detangle’ the sample into individual lineages and there are limits to the accuracy of such tools. For example, a benchmarking study which included Alcov concluded that calls at frequencies less than 5% should be interpreted with caution due to high background noise in WW samples^43^. As a result, in municipal WW surveillance, patterns and trends in data, especially in slowly emerging lineages, lend confidence to the predictions. Initial detections in this study were predicted at 5% or higher (apart from BA.2.75 and BQ*), but there are other factors that can result in lower confidence in single-point signal spikes, such as the possibility that diagnostic mutations were missed. For this reason, in the cases (BQ*, XBB.1.5*) where the initial detection was more than 2 weeks ahead of any other detections considered in this study (other WW collected from the sites included in this analysis or Ontario clinical) the second detections should be considered as well when calculating lead time gained (bracketed values in Table 2). If WW sequencing in airports were to be used to inform pathogen impact on public health and inform policy changes in future pandemics, recommendations from data collectors to the recipients of the data would have to be well balanced to account for differences between single and multiple detections with corresponding interpretations weighted accordingly.

It is valuable as well to be able to track the potential national entry of pathogens and their emergence in the community through airport coupled with municipal WW surveillance amidst limited clinical testing to inform how detections at entry points translate to transmission in the surrounding regions^35^. The long timespan between terminal and municipal WW detections in some instances (e.g. BQ*, Fig. 2C) suggests that in some cases variants may not have been quick to spread after initial introduction to the local population, or that they were present in airport WW due to transitioning travelers. The transmissibility and infection rates of SARS-CoV-2 differ due to many factors (i.e. by variant, age demographics, and vaccination rates of individuals), and not all viral detections directly translate to continued transmission^56^. After initial detection of emerging lineages, the signal in Toronto Pearson WW generally continued to fluctuate, with higher peaks in frequency than the municipal WW from the surrounding regions. Toronto Pearson WW then would not be useful to monitor subsequent local trends in VOC dominance past the initial community introduction, which has been the key role of municipal WW testing in SARS-CoV-2 surveillance.

### Unique mutations influence the ability to accurately call specific sub-lineages

XBB lineages provided an example of how frequency predictions using WW sequencing in this study were affected by availability of unique mutations, despite the ability of prediction tools including Alcov to use shared mutations as well to make lineage predictions. XBB*, as a recombinant of BA.2.10.1 and BA.2.75 sub-lineages (BJ.1 and BM.1.1.1 respectively), had a distinct mutation constellation compared to BQ* and BF* lineages, which descended from BA.5 and other BA.5 sub-lineages which were in circulation around the time of XBB* emergence in Ontario^47,57^. Despite high transmissibility and associated rapid spread, XBB* was detected in Toronto Pearson WW a week ahead of the first clinical case^48^. XBB.1.5 had only three nucleotide mutations (C44T, T17124C, T23018C) differentiating it from XBB.1, with only one of these resulting in an amino acid change (T23018C yields S: F486P), making it less distinct for de-mixing analyses^33^. WW data collected from Toronto Pearson was unable to provide more than one early detection compared to clinical sequencing in the case of XBB.1.5. This is likely because Alcov was unable to confidently and specifically predict XBB.1.5 when it was present in samples at low abundance due to similarity to other XBB* lineages. XBB.1.5* was also prevalent in the USA before emergence in Ontario so it is likely that early transmission occurred across the Canada-USA border land crossings, which would affect lead time^58^. XBB.1.16* had 12 nucleotide changes (C11750T, C11956T, T12730A, A14856G, G18703T, A22101T, C22995G, T23018C, G27915T, T28297C, A28447G, C29386T) compared to XBB.1, and XBB.1.9* had five nucleotide changes (G5720A, C12789T, T23018C, G27915T, T28297C) compared to XBB.1 which made these two groups of XBB sub-lineages slightly easier to differentiate and thus detect in WW (Table 2)^33^. In addition to the differences in number of unique mutations, viral shedding across lineages/sublineages may not be the same^59^. For example, one lineage might be shed for a longer period than other lineages. This in turn might affect the proportion and amplification of new variants in WW^59^.

### Terminal and aircraft sewage are most valuable when used together

Only Terminal 1 and 3 WW provided an average lead time prior to the PHO initial clinical detection. We would expect initial detection of emerging lineages which originate outside of Canada to be in the pooled aircraft sewage more often due to the contributing populations being more transient, but in this study emerging VOCs were more frequently detected in terminal WW first. The pooled aircraft sewage was collected using passive samplers from January 1 until October 21, 2022, much longer than the Terminals were, and many of the VOCs in this study emerged before October 2022. While passive samplers were being used, the coverage for pooled aircraft sewage samples was lower than after the auto sampler was installed (Supplementary Table S3), which could be contributing to the lack of lead time, though the median BOC was lower in the terminals than in the passive sampler pooled aircraft samples. The later detection and lack of average lead time of many lineages by pooled aircraft WW is also confounded by a possible reluctance of passengers to use the airplane lavatory, in favor of waiting until landing at the airport, or that the lavatories may not be needed on shorter flights^60^. After October 21st 2022 when an autosampler was used for pooled aircraft sewage as well, BOC was on average much higher than in Terminals 1 and 3 (Supplementary Table S3) likely due to the triturator sewage being less dilute. In the examples of two XBB sub-lineages, XBB.1.9 and XBB.1.16 (Fig. 2f and d, supplementary Figures S6 and S7), which emerged in Ontario after the switch in collection method, the pooled aircraft sewage samples had earlier signals and in the case of XBB.1.16 provided the only early detection. On the other hand, in the example of the S: R346T mutation, the first detection in pooled aircraft sewage occurred within the same week as terminal first detections even though this was during the period when passive samplers were in use at the triturator. Additionally, the first detection of BF* in Terminals was in a sample collected using a passive sampler, yet it still resulted in a lead time (Supplementary Table S1, Fig. 2b). Whether the differences in performance between the two sample types were due to the timing of sampling method change or dilution of the sample by local staff members, both sample types will be valuable to collect in future pandemic preparedness surveillance programs as they provide insight into different population types.

### Effect of sampling method, sequencing methods and sampling site

There are variations in how samples were processed in this study due to the collaboration of three sequencing laboratories to generate data for sub-sets of sampling sites. BOC was analyzed and compared in Supplementary Table S3. More of the locations along the SARS-CoV-2 genome where defining mutations could be found were captured in sequence data with higher BOC, which aided in detecting and distinguishing lineages. All sample groups had median BOC greater than 75% of the genome so all sites had many samples with close to complete genomes. If method, rather than sampling site, was influencing first detection location it would be expected that the University of Guelph Pooled Aircraft samples would detect VOCs first most often due to higher BOC, but this was not observed. While municipal WW did have lower average BOC all-together and first detections did not occur in municipal WW during this study, the Peel region average BOC was slightly higher than the average BOC for Terminals 1 and 3 of Toronto Pearson, which had the most frequent first detections. Samples processed by the lab at University of Waterloo provided an opportunity to analyze how the same methods yielded different results for different sampling locations as well, with average BOC differing between regions.

It is possible that the differences in sample extraction methods biased the fragments of SARS-CoV-2 viral RNA that were captured prior to ARTIC amplification and sequencing. The sequencing labs which contributed data to this study from University of Guelph and University of Waterloo used Ceres Nanotrap Microbiome A Particles to capture viral particles and other microbes from the supernatant fraction of the WW while Western University sequencing lab pelleted the sample by centrifugation. A study comparing extraction methods found that Ceres Nanotrap beads and centrifugation to extract from the solids pellet had similar human viral read yields^61^. The same study also found that while the centrifugation method had higher RNA yields and longer fragments than nanotrap beads, the centrifugation method did not produce complete human viral genomes from sequencing after probe-capture enrichment^61^. In this study the Toronto region had the lowest average BOC which is consistent with these findings though using tiled amplicon sequencing likely reduced the differences observed between extraction methods. Additionally, the Qiagen RNA extraction kit used at University of Guelph was designed for Viral RNA capture while the University of Waterloo and Western University use a more general RNA extraction kit. This could have had an influence on BOC, as samples processed through University of Guelph had higher overall BOC.

### Target selection as a limitation

Pandemic preparedness through WW surveillance at airports will also require knowledge of a potentially emerging virus prior to its arrival in Canada. In the current study, tiled amplicons designed specifically to capture only SARS-CoV-2 were used to sequence the SARS-CoV-2 particles found in WW. This strategy works well for catching emerging VOCs because once the amplicons have been sequenced, the assembled genomes can be re-analyzed looking for new lineages retrospectively as new VOCs become concerning in other parts of the world. Even so, this strategy cannot identify new lineages. New lineage prediction could be accomplished using bioinformatics approaches such as non-negative matrix factorization (NMF) in the future^62^.

WW surveillance and clinical lineage reporting by GISAID or PHO reports also depend on lineage definition availability. If lineages are introduced into a community via infected individuals shortly after the initial evolution of the lineage, a lack of defined mutation constellation or lineage definition can delay identification of the lineage and therefor data availability. While clinical sequences, if near-complete, can contribute to efforts to define new lineages, WW sequencing cannot due to the complex nature of the viral community it captures.

If the surveillance is extended to other viral targets, sequencing strategies will have to be developed for each different target ahead of their potential spread into Canada for the data to be available early enough to serve as an early warning for public health decision making. This could mean developing and implementing more tiled assays for virus families that are an ongoing concern (RSV, Influenza) or those that have caused outbreaks in recent history i.e. Mpox^63^. Alternatively, deep shotgun metagenomic sequencing could be performed to collect data without a target for detection of potentially novel viral threats. It should be noted that shotgun sequencing of viral RNA is resource-intensive as viral reads are often less abundant than bacterial reads, requiring a large amount of sequencing space and/or rigorous pre-selection or rRNA depletion to capture the viral community in detail^25,26^. RT-qPCR has been used to detect a wider panel of pathogens in aircraft and airport terminal WW and could be used to detect a known, pre-determined set of pathogens in future surveillance programs, but would be limited by assay development if used to follow virus evolution^23^.

### Turn-around time to reporting as a limitation

During data collection for this project, results of sequencing for airport sampling sites were reported to airport personnel, MECP, and PHAC on a bi-weekly basis but not to surrounding municipalities due to privacy agreements while piloting the Toronto Pearson WW sampling program. It is important to note the turn-around time of sequencing from sample receipt to reporting is 1–2 weeks. This means that in the above examples if data were available to municipalities or in a centralized hub, the lead time to react to a first detection with policy decisions in preparation for a new VOC import after notification by the data collectors would be less than the lead times given in Tables 2 and 4 which are based on the sample collection dates. In the case of detections that occurred only a week prior to the clinical detection, the notification would not precede testing of infected individuals, but the data would at least be available to the public health unit that the VOC is present earlier than clinical reports.

Once a clinical sample was collected which would be reported as a given lineage, there was a delay of 1–4 weeks before sequencing data from the clinical sampling for that case was made available to the PHUs^50,51^. The GISAID sequences used in this study were reported 2 to 8 weeks after collection (Table 3) but had been collected earlier than WW first detects of these lineages making these GISAID clinical sequences a possible valuable resource, though not all variants were available for searching on this database (Table 4)^28^. Data-availability for clinical sequencing in both cases was dependent on multiple parties (clinical practitioners) to report which could cause inconsistency in reporting times.

After a variant is introduced into a community/region, it is valuable information for the PHUs to be made aware of, so the reporting turnaround times of WW surveillance in general are valuable while PHUs await clinical testing results. Airport WW surveillance reports would be an even earlier datapoint to inform PHUs of emerging lineages even if the lead time to the variant’s detection in the region by clinical testing is not very long.

## Conclusion

WW collection at Toronto Pearson provided an example of the early warning capabilities of WW surveillance at points of entry into Canada. In this study an average lead time of 4 weeks was observed between the first detection of an emerging SARS-CoV-2 lineage in WW collected from Toronto Pearson Terminals 1 and 3 and the first Ontario clinical detection of the same lineage, with lead times of 1 and 3 weeks provided by pooled aircraft sewage in later emerging lineages (XBB.1.9* and XBB.1.16*). This was always a longer lead time than between municipal WW and clinical detections. WW surveillance at points of entry such as Toronto Pearson could provide an early warning signal for pathogen entry into surrounding regions, contingent on turnaround time to reporting. This study demonstrates early detection by amplicon sequencing of SARS-CoV-2 from WW. However, it should be emphasized that similar strategies could be used to establish surveillance programs for other pathogen targets, and human health concerns such as antimicrobial resistance. WW surveillance at points of entry such as airports should be considered as a core tool when designing programs for pandemic preparedness.

## Electronic supplementary material

Below is the link to the electronic supplementary material.

Supplementary Material 1

Supplementary Material 2

Supplementary Material 3

## Data Availability

The raw sequencing data can be viewed on the Sequence Read Archive (SRA) under bio project PRJNA1088471.
