# Supplementary material for: Genomic surveillance of Canadian airport wastewater samples allows early detection of emerging SARS-CoV-2 lineages
Source: Sci Rep. 2024 Nov 3;14:26534. doi: 10.1038/s41598-024-76925-6 (PMC11532424; doi:10.1038/s41598-024-76925-6)
Supplement: Supplementary file 3 — Supplementary Material 3 [file 41598_2024_76925_MOESM3_ESM.pdf]

Genomic Surveillance of Canadian Airport Wastewater Samples Allows Early Detection of Emerging SARS-CoV-2 Lineages

Alyssa K. Overton[1 *], Jennifer J. Knapp[1], Opeyemi U. Lawal[2], Richard Gibson[3], Anastasia A. Fedynak[2], Adebowale I. Adebiyi[3], Brittany Maxwell[4], Lydia Cheng[5], Carina Bee[7], Asim Qasim[7], Kyle Atanas[5], Mark Payne[7], Rebecca Stuart[6], Manon D. Fleury[8], Natalie C. Knox[8], Delaney Nash[1,9], Yemurayi C. Hungwe[1], Samran R. Prasla[1], Hannifer Ho[1], Simininuoluwa O. Agboola[1], Su-Hyun Kwon[1], Shiv Naik[1], Valeria R. Parreira[2], Fozia Rizvi[2], Melinda J. Precious[2], Steven Thomas[4], Marcos Zambrano[4], Vixey Fang[7], Elaine Gilliland[5], Monali Varia[5], Maureen Horn[5], Chrystal Landgraff[8], Eric J. Arts[3], Lawrence Goodridge[2], Devan Becker[10], Trevor C. Charles[1,9 *]

[1]University of Waterloo

[2]University of Guelph

[3]Western University

[4]Greater Toronto Airports Authority

[5]Regional Municipality of Peel

[6]Toronto Public Health

[7]York Region Public Health

[8]Public Health Agency of Canada

[9]Metagenom Bio Life Science Inc.

[10]Wilfrid Laurier University

Supplementary Figures S1-S7, Supplementary table S3 and Supplementary methods

Supplementary Figures and Tables

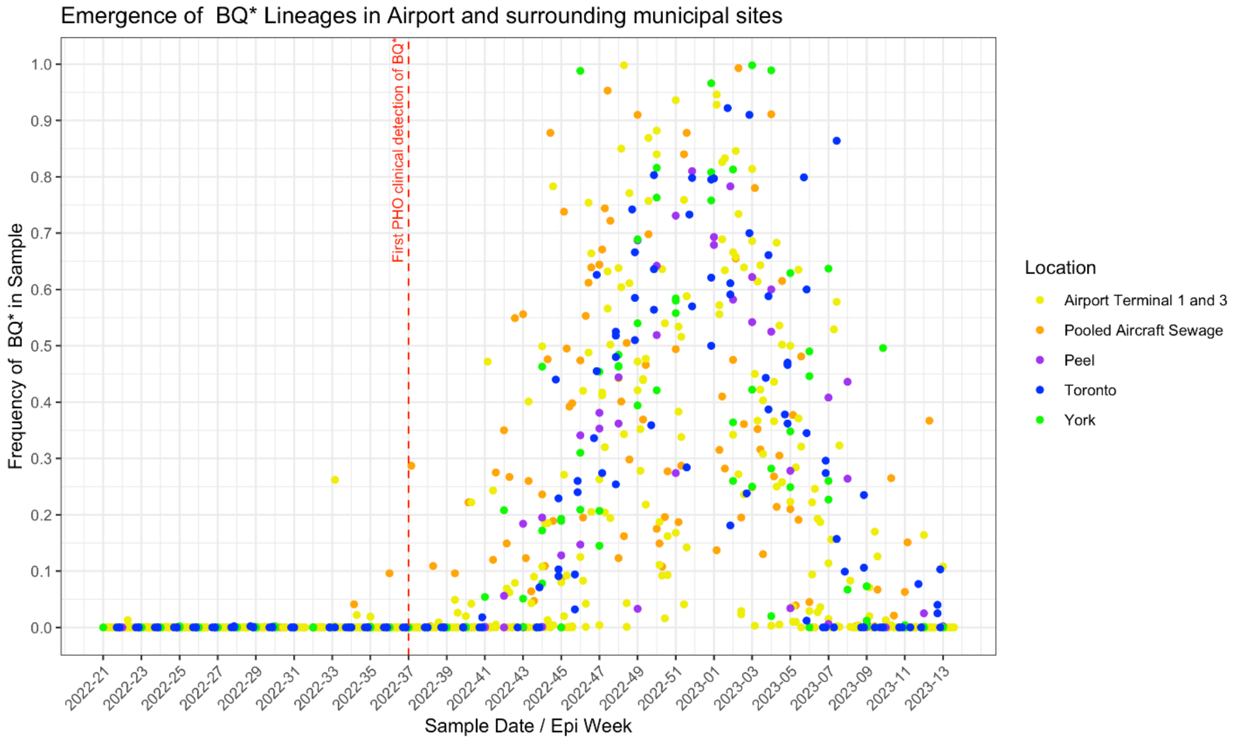

Supplementary Figure S1. Frequency of BQ lineages in WW samples from Toronto Pearson and surrounding municipal sites in Toronto, York, and Peel regions. Each point represents a single WW sample. Red dashed line represents the date of the first clinical sequence for BQ* in Ontario from PHO reports.*

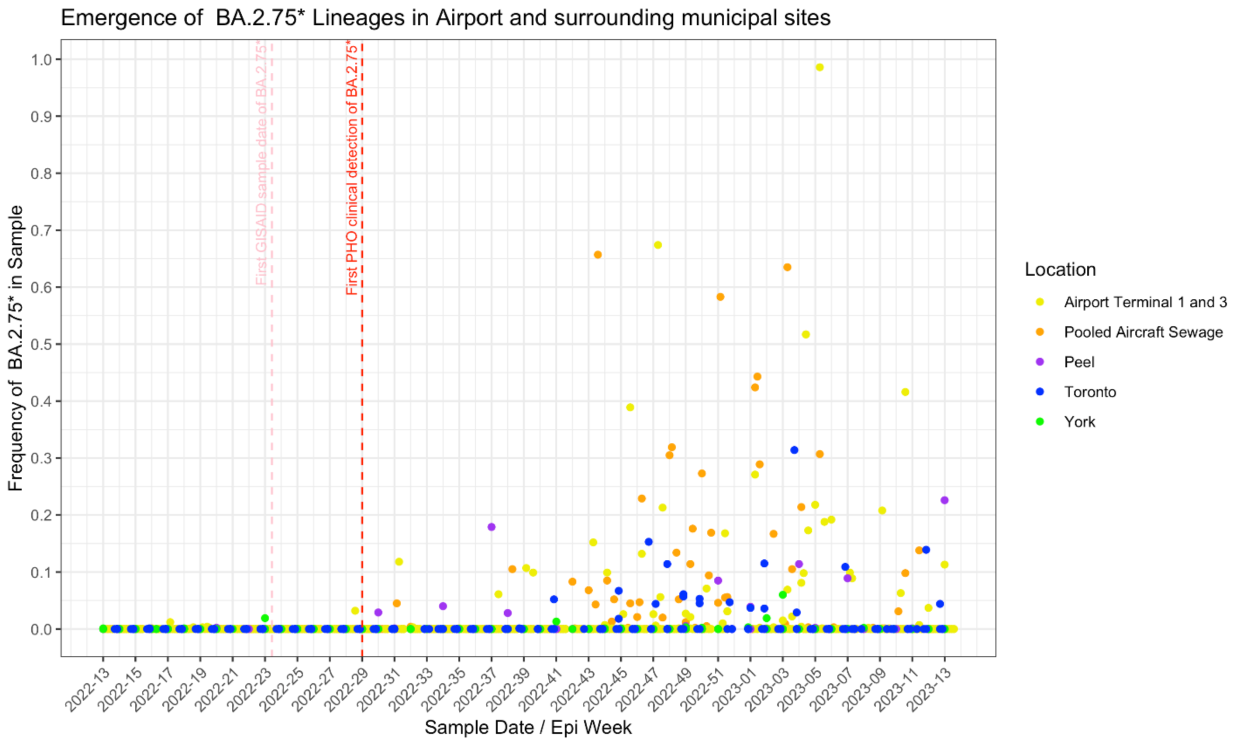

Supplementary Figure S2. Frequency of BA.2.75 lineages in WW samples from Toronto Pearson and surrounding municipal sites in Toronto, York, and Peel regions. Each point represents a single WW sample. Red dashed line represents the date of the first clinical sequence for BA.2.75* in Ontario from PHO reports. Pink dashed line represents the date of the first clinical sequence for BA.2.75 available in GISAID.*

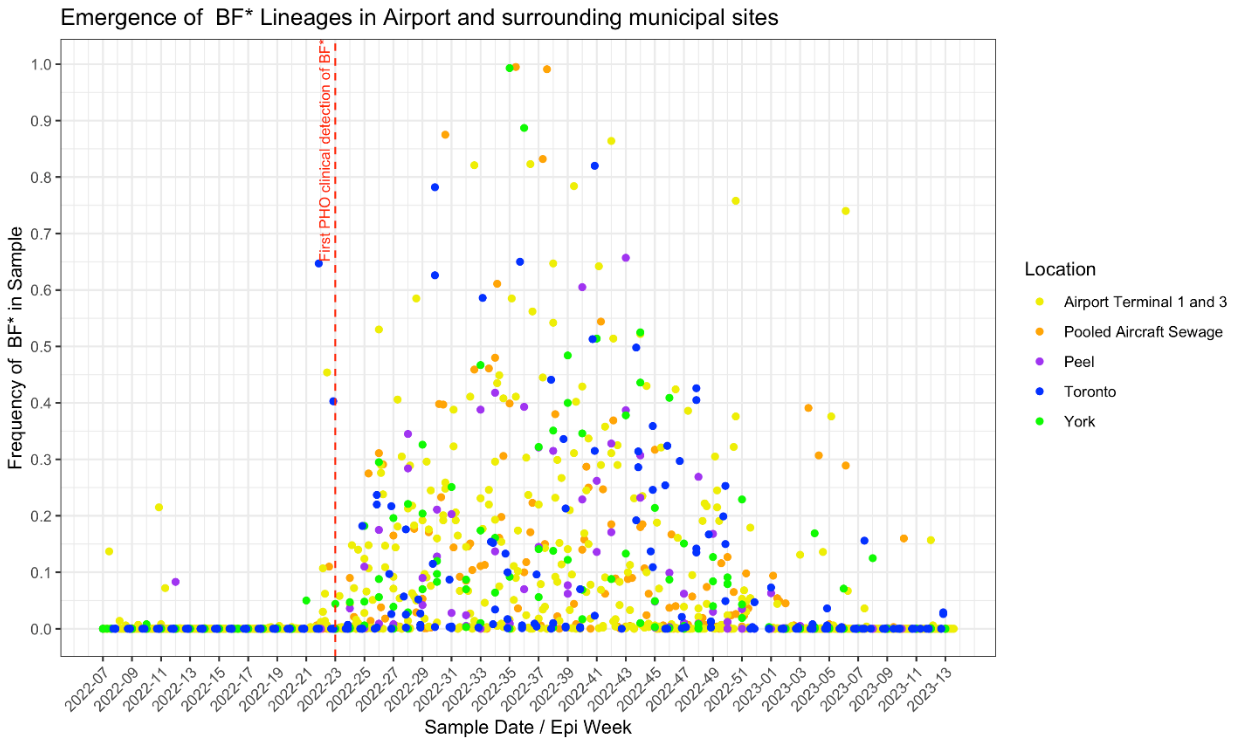

Supplementary Figure S3. Frequency of BF lineages in WW samples from Toronto Pearson and surrounding municipal sites in Toronto, York, and Peel regions. Each point represents a single WW sample. Red dashed line represents the date of the first clinical sequence of BF* in Ontario from PHO reports.*

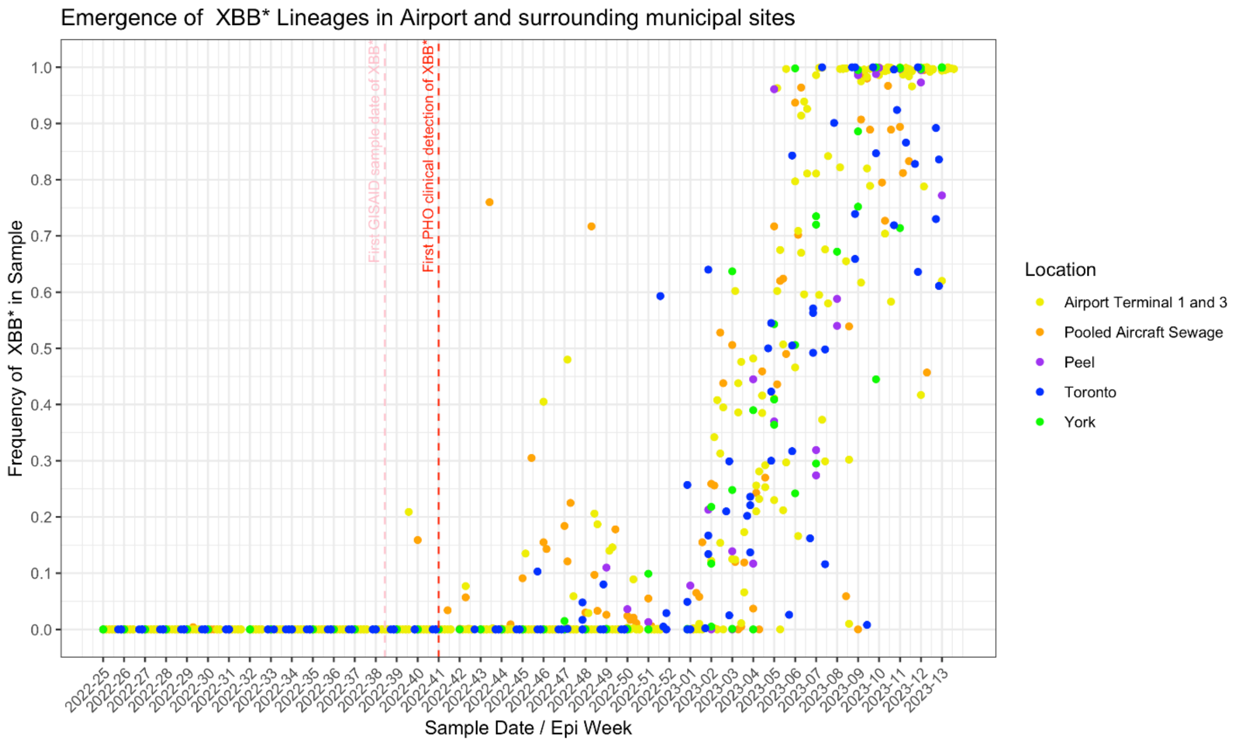

Supplementary Figure S4. Frequency of XBB lineages in WW samples from Toronto Pearson and surrounding municipal sites in Toronto, York, and Peel regions. Each point represents a single WW sample. Red dashed line represents the date of the first clinical sequence of XBB* in Ontario from PHO reports. Pink dashed line represents the date of the first clinical sequence for XBB* available in GISAID.*

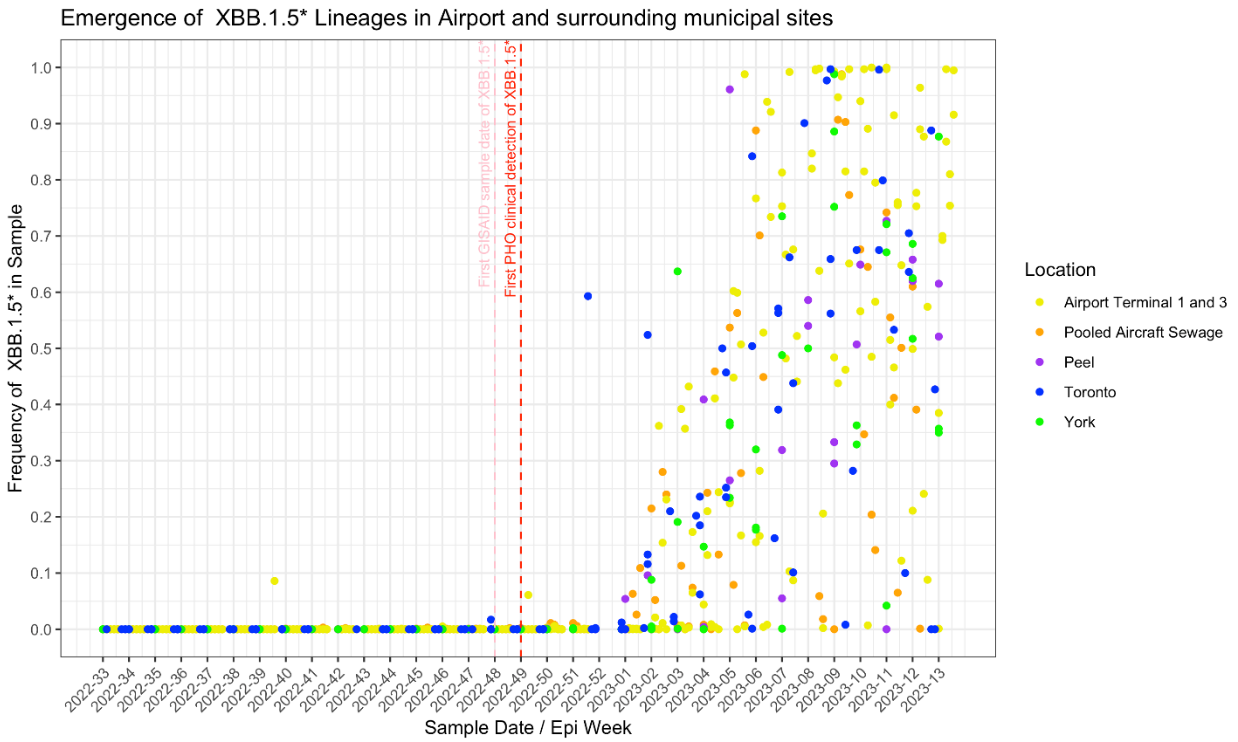

Supplementary Figure S5. Frequency of XBB.1.5 lineages in WW samples from Toronto Pearson and surrounding municipal sites in Toronto, York, and Peel regions. Each point represents a single WW sample. Red dashed line represents the date of the first clinical sequence of XBB.1.5* in Ontario from PHO reports. Pink dashed line represents the date of the first clinical sequence for XBB.1.5* available in GISAID.*

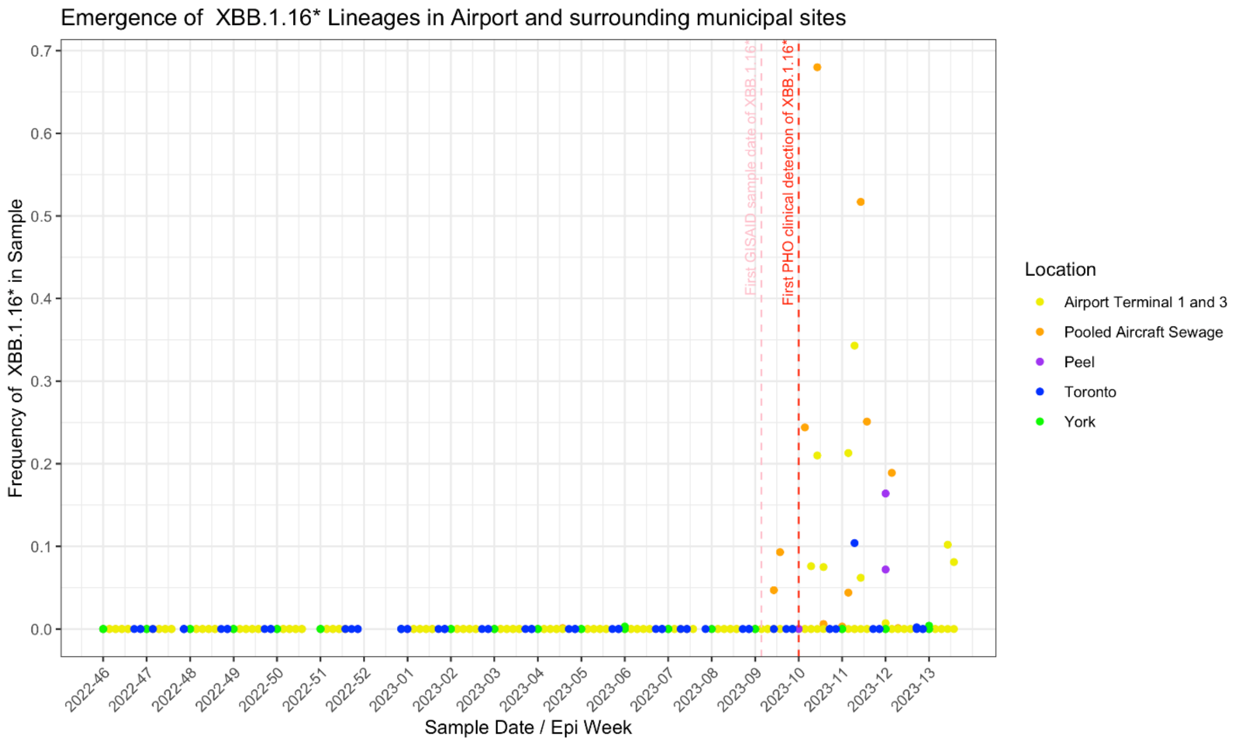

Supplementary Figure S6. Frequency of XBB.1.16 lineages in WW samples from Toronto Pearson and surrounding municipal sites in Toronto, York, and Peel regions. Each point represents a single WW sample. Red dashed line represents the date of the first clinical sequence of XBB.1.16* in Ontario from PHO reports. Pink dashed line represents the date of the first clinical sequence for XBB.1.16* available in GISAID.*

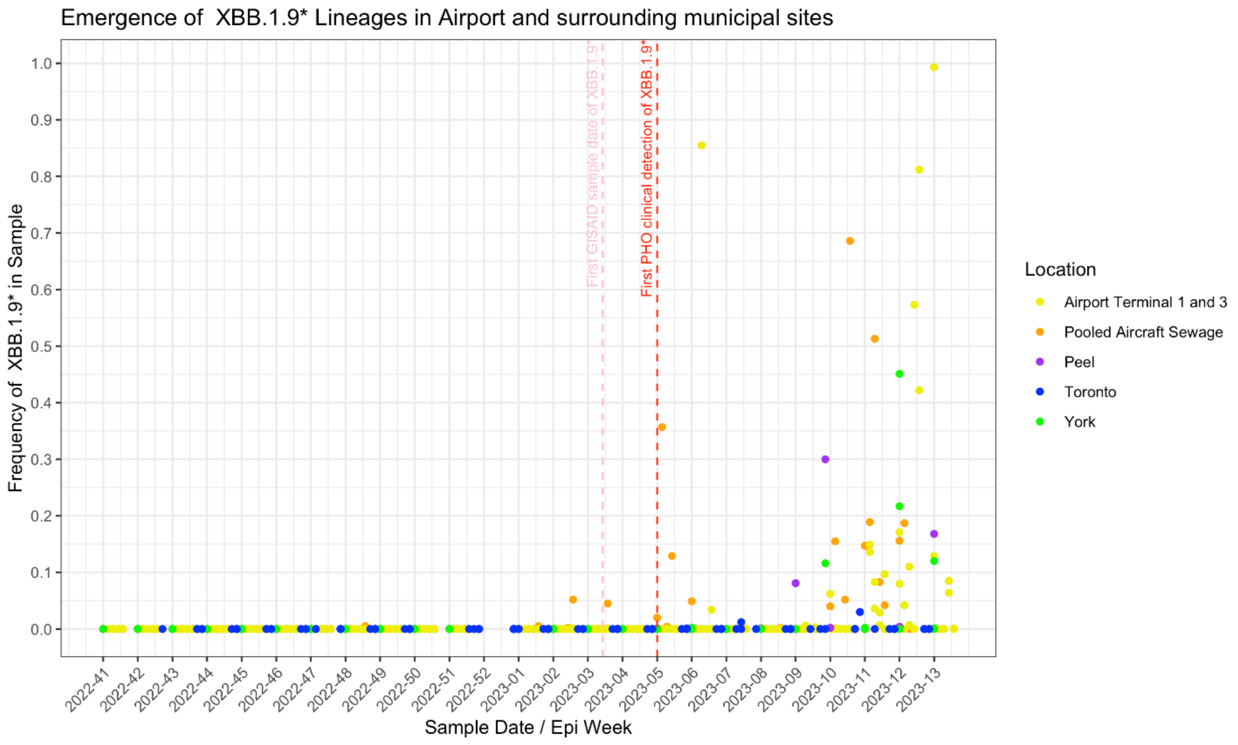

Supplementary Figure S7. Frequency of XBB.1.9 lineages in WW samples from Toronto Pearson and surrounding municipal sites in Toronto, York, and Peel regions. Each point represents a single WW sample. Red dashed line represents the date of the first clinical sequence of XBB.1.9* in Ontario from PHO reports. Pink dashed line represents the date of the first clinical sequence for XBB.1.9* available in GISAID.*

Supplementary Tables S1 and S2 are attached as spreadsheets

Supplementary Table S3: Breadth of Coverage Analysis

Sample	SequencingPartner	AverageBOC	Median BOC	st dev
**Region**				
Terminal 1 and 3	Waterloo	76.55423729	82	18.38826839
Pooled Aircraft	Guelph	80.87124464	96	27.17438024
York	Waterloo	71.46892655	76	20.65701603
Peel	Waterloo	77.07142857	83.5	19.06012982
Toronto	Western	66.75618375	76	25.45294117
**Terminal**				
Terminal 1		75.35932203	80	18.03216635
Terminal 3		77.74915254	84	18.66124755
**Pooled Aircraft Sample Method**				
Aircraft passive sampler		73.07801418	90	30.11492665
Aircraft auto sampler		92.81521739	98	15.63714179
**Municipal Sample Method**				
Municipal Grab		74.45132743	79	19.93041
Municipal Composite 24hr		69.42917548	78	23.75998187
**Sequencing Partner**				
	Waterloo	75.61659193	81	19.07687053
	Guelph	80.87124464	96	27.17438024
	Western	66.75618375	76	25.45294117

Table showing average, median and st dev of BOC > 10 data broken into different sample groups as indicated by sub-headers for comparison. All sequenced samples were included even if they were discarded from lineage calling analysis.

Supplementary Methods

Sum Frequencies for Lineages - Python

```
import pandas as pd
import json

# Load the alias key
with open('/Users/jennk/Documents/Data_Chales_Lab/paper_airport/alias_key.json', 'r') as f:
    alias_key = json.load(f)

# Load the CSV file
csv_file_path = '/Users/jennk/Documents/Data_Chales_Lab/paper_airport/all_samples_lineages.csv'
df = pd.read_csv(csv_file_path)
```

```
# Long form names for specified lineages
lineages_of_interest = {
    'BQ': 'B.1.1.529.5.3.1.1.1.1',
    'BA.2.75': 'B.1.1.529.2.75',
    'BF': 'B.1.1.529.5.2.1',
    'XBB': 'XBB',  # Special handling required
    'XBB.1.5': 'XBB.1.5',
    'XBB.1.16': 'XBB.1.16',
    'XBB.1.9': 'XBB.1.9'
}

# Function to get all child lineages for a given lineage
def get_subset_keys(dictionary, start_string):
    return {key for key, value in dictionary.items() if isinstance(value, str) and
value.startswith(start_string) or isinstance(value, list) and any(isinstance(item, str) and
item.startswith(start_string) for item in value)}

lineage_results = {}

for lineage, start_string in lineages_of_interest.items():
    lineage_keys = get_subset_keys(alias_key, start_string)
    lineage_keys.add(lineage)  # Include the lineage itself
    lineage_results[lineage] = lineage_keys

# Exclude XBF from BF lineage
lineage_results['BF'] = {key for key in lineage_results['BF'] if not key.startswith('XBF')}

# Initialize dictionary to store lineage columns
lineage_columns = {lineage: set() for lineage in lineage_results.keys()}

# Identify columns matching each lineage and its children
for lineage, child_keys in lineage_results.items():
    for col in df.columns:
        col_lineages = col.split(' or ')
        if all(any(child_key in lineage_part for child_key in child_keys) for lineage_part in col_lineages):
            lineage_columns[lineage].add(col)

# Debugging: print identified columns for each lineage
for lineage, columns in lineage_columns.items():
    print(f"{lineage}: {columns}")

# Function to sum columns for each lineage and round to 3 decimal places
def sum_lineage_columns(df, lineage, columns):
    if columns:
        df[f'{lineage}_summary'] = df[list(columns)].sum(axis=1).round(3)
```

```
    else:
        df[f'{lineage}_summary'] = 0

# Sum the columns and create summary columns
for lineage, columns in lineage_columns.items():
    sum_lineage_columns(df, lineage, columns)

# Reorder columns to insert summary columns after 'BreadthOfCoverage'
summary_columns = [f'{lineage}_summary' for lineage in lineages_of_interest]
cols = list(df.columns)
for col in summary_columns:
    cols.insert(3, cols.pop(cols.index(col)))

df = df[cols]

# Save the modified DataFrame back to a CSV file
output_csv_file_path =
'/Users/jennk/Documents/Data_Chales_Lab/paper_airport/summary_all_samples_lineages.csv'
df.to_csv(output_csv_file_path, index=False)
```

Data Preparation for Plots

```
suppressPackageStartupMessages({
    library(readxl)
    library(dplyr)
    library(tidyr)
    library(ggplot2)
    theme_set(theme_bw())
    library(lubridate)
})
### NOT RUN
# Processing script to remove unused columns and
# give columns standardized names.

# This was run once then copied here for posterity.

air1 <- read_excel("summary_all_samples_lineages.xlsx")
air <- air1[, 1:10]
names(air) <- c("Sample_Name", "Sample_Date", "Breadth_of_Coverage",
    "XBB.1.9*", "XBB.1.16*", "XBB.1.5*",
    "XBB*", "BF*", "BA.2.75*", "BQ*")

air <- air %>%
    mutate(Location = gsub("(20\\d+)|(-\\d+)", "", Sample_Name)) %>%
    mutate(Location = ifelse(
```

```
        Location %in% c("Ashbridges", "AshbridgesBay", "AshbridgeBay"),
        yes = "Ashbridges",
        no = ifelse(
          Location %in% c("HighlandCreek", "Highland", "HighlandCR"),
          yes = "HighlandCreek",
          no = Location)
    )) %>%
    mutate(Group = case_when(
        Location %in% c("Humber", "Ashbridges", "HighlandCreek", "NorthToronto") ~ "Toronto",
        Location %in% c("As", "At") ~ "Pooled Aircraft Sewage",
        Location %in% c("A1", "A3") ~ "Airport Terminal 1 and 3",
        Location %in% c("P1", "P2") ~ "Peel",
        Location %in% c("Y1", "Y5", "Y6") ~ "York",
        TRUE ~ "Devan Missed One"
    ))
air <- select(air, -Sample_Name) %>%
    pivot_longer(cols = !c(Group, Location, Sample_Date, Breadth_of_Coverage),
        values_to = "Frequency", names_to = "Lineage") %>%
    mutate(Sample_Date = ymd(Sample_Date),
        Frequency = ifelse(Frequency > 1, 1, Frequency))
write.csv(air, file = "summary_all_samples_clean.csv")
rm(air1)
### END NOT RUN
air <- read.csv("summary_all_samples_clean.csv") %>%
    filter(Breadth_of_Coverage >= 20)

gisaid <- data.frame(
    Lineage = c("BQ*", "BA.2.75*", "BF*", "XBB*",
        "XBB.1.5*", "XBB.1.16*", "XBB.1.9*"),
    Gisaid_Sample = ymd(c("", "2022-06-09", "", "2022-09-22",
          "2022-11-28", "2023-02-28", "2023-01-19")),
    Gisaid_Report = ymd(c("", "2022-06-21", "", "2022-10-03",
          "2022-12-12", "2023-04-24", ""))
)
pho <- data.frame(
    Lineage = c("BQ*", "BA.2.75*", "BF*", "XBB*",
        "XBB.1.5*", "XBB.1.16*", "XBB.1.9*"),
    Pho_Week = c(37, 29, 23, 41,
        49, 52 + 10, 52 + 5) + 52
)

date_seq <- seq(ymd("2022-01-01"), ymd("2024-12-31"), 1)
epiweeks <- data.frame(
    EpiWeek = epiweek(date_seq) + 52 * (year(date_seq) - 2021),
```

```r
  date = date_seq)
epiweeks <- epiweeks[epiweeks$EpiWeek %in% pho$Pho_Week, ]

air2 <- left_join(air, gisaid, by = "Lineage") %>%
  mutate(Lead_Sample = as.numeric(ymd(Sample_Date) - ymd(Gisaid_Sample)),
    Lead_Report = as.numeric(ymd(Sample_Date) - ymd(Gisaid_Report)),
    Epi_Week = 52 * (year(Sample_Date) - 2021) + epiweek(Sample_Date)) %>%
  left_join(pho, by = "Lineage") %>%
  mutate(Lead_Epi = Epi_Week - Pho_Week) %>%
  mutate(Detection = Frequency >= 0.01) %>%
  mutate(Group = ordered(Group, levels = levels(factor(Group))[c(1,3,4,5,2)]))

air4 <- air2 %>%
  filter(Lead_Epi > -17) %>%
  group_by(Lineage, Group) %>%
  arrange(Sample_Date) %>%
  mutate(`Freq >= 0.01` = cumsum(Frequency > 0.01),
    `Freq >= 0.05` = cumsum(Frequency > 0.05)) %>%
  pivot_longer(cols = c(`Freq >= 0.01`, `Freq >= 0.05`))


air5 <- air2 %>%
  filter(Lead_Epi > -17) %>%
  group_by(Lineage, Group) %>%
  arrange(Sample_Date) %>%
  mutate(`Freq >= 0.01` = cumsum(Frequency > 0.01),
    `Freq >= 0.05` = cumsum(Frequency > 0.05)) %>%
  pivot_longer(cols = c(`Freq >= 0.01`, `Freq >= 0.05`))
# https://stackoverflow.com/questions/54438495/shift-legend-into-empty-facets-of-a-faceted-plot-in-ggplot2
shift_legend3 <- function(p) {
  pnls <- cowplot::plot_to_gtable(p) %>% gtable::gtable_filter("panel") %>%
    with(setNames(grobs, layout$name)) %>%
    purrr::keep(~identical(.x, zeroGrob()))

  if (length(pnls) == 0) stop("No empty facets in the plot")

  lemon::reposition_legend(p, "center",
    panel = names(pnls))
}
```

Frequency Plots (log scale)

```r
g <- ggplot(filter(air2, Lead_Epi > -17, Lead_Epi < 10, Frequency > 0)) +
  theme_bw() +
```

```
   aes(x = Lead_Epi, y = Frequency, colour = Group) +
   geom_point(size = 1, mapping = aes(alpha = Detection)) +
   scale_alpha_manual(values = c(0.4, 1)) +
   facet_wrap(~ Lineage, ncol = 2) +
   geom_vline(xintercept = 0) +
   geom_hline(yintercept = c(0.01), col = "grey", linetype = 2) +
   geom_hline(yintercept = c(0.05), col = "grey", linetype = 2) +
   labs(x = "Epiweeks to/from First Clinical Case",
     y = "Relative Demixing Frequency of Lineage from Alcov (log scale)",
     title = "", colour = NULL) +
   guides(alpha = "none") +
   scale_y_log10() +
   scale_colour_brewer(palette = "Dark2")
 shift_legend3(g)
```

Cumulative Plots

```
g <- filter(air4, Lead_Epi > -20, Lead_Epi < 10)  %>%
   mutate(name = ordered(name, levels = rev(levels(factor(name))))) %>%
   ggplot() +
   theme_bw() +
   geom_vline(xintercept = 0, colour = "darkgreen", linewidth = 1.25) +
   aes(x = Lead_Epi, y = value, colour = Group, linetype = name) +
   geom_step() +
   facet_wrap(~ Lineage, ncol = 2) +
   coord_cartesian(ylim = c(0, 10)) +
   labs(x = "Epiweeks Since First Clinical Case",
     y = "Cumulative Detections",
     linetype = "Detection Threshold",
     colour = "Location") +
   scale_y_continuous(minor_breaks = 0:10, breaks = seq(0, 10, 2)) +
   scale_x_continuous(minor_breaks = -20:20, breaks = seq(-20, 20, 2)) +
   scale_colour_brewer(palette = "Dark2") +
   theme(legend.box = "horizontal")

 shift_legend3(g)
```

Summary Statistics

```
air4 %>%
   filter(Lead_Epi > -20, value == 1) %>%
   mutate(Detection_Threshold = case_when(
     Detection ~ 0.01,
     TRUE ~ 0.05
   )) %>%
   group_by(Group, Detection_Threshold) %>%
   summarise(
     earliest_first_date = min(Lead_Epi),
```

```
    mean_first_date = mean(Lead_Epi),
    sd_first_date = sd(Lead_Epi)
  ) %>%
  arrange(Group, Detection_Threshold) %>%
  knitr::kable()
```

Summary Statistics GISAID

```
air5 %>%
  filter(Lead_Epi > -20, value == 1) %>%
  mutate(Detection_Threshold = case_when(
    Detection ~ 0.01,
    TRUE ~ 0.05
  )) %>%
  group_by(Group, Detection_Threshold) %>%
  summarise(
    earliest_first_date = min(Lead_Sample, na.rm = TRUE),
    mean_first_date = mean(Lead_Sample, na.rm = TRUE),
    sd_first_date = sd(Lead_Sample, na.rm = TRUE)
  ) %>%
  arrange(Group, Detection_Threshold) %>%
  knitr::kable()
```

Supplementary Scatter Plots - R

```
suppressPackageStartupMessages({
  library(readxl)
  library(dplyr)
  library(magrittr)
  library(tidyr)
  library(ggplot2)
  theme_set(theme_bw())
  library(lubridate)
  library(epitools)
})

air <- read.csv("summary_all_samples_clean.csv") %>%
  filter(Breadth_of_Coverage >= 20)

gisaid <- data.frame(
  Lineage = c("BQ*", "BA.2.75*", "BF*", "XBB*",
              "XBB.1.5*", "XBB.1.16*", "XBB.1.9*"),
  Gisaid_Sample = ymd(c("", "2022-06-09", "", "2022-09-22",
                        "2022-11-28", "2023-02-28", "2023-01-19")),
  Gisaid_Report = ymd(c("", "2022-06-21", "", "2022-10-03",
                        "2022-12-12", "2023-04-24", ""))
)
```

```
pho <- data.frame(
  Lineage = c("BQ*", "BA.2.75*", "BF*", "XBB*",
              "XBB.1.5*", "XBB.1.16*", "XBB.1.9*"),
  Pho_Week = c(37, 29, 23, 41,
               49, 10, 5),
  Year = c(2022, 2022, 2022, 2022, 2022, 2023, 2023)
)

# Convert Pho_Week and Year to dates
pho <- pho %>%
  mutate(Pho_Date = as.Date(paste(Year, Pho_Week, 1, sep = "-"), "%Y-%U-%u"))

# Ensure Sample_Date is a Date object and add Epiweek and Year columns
air <- air %>%
  mutate(Sample_Date = as.Date(Sample_Date),
         Epiweek = as.numeric(as.week(Sample_Date)$week),
         Year = year(Sample_Date),
         Epiweek_Year = paste(Year, sprintf("%02d", as.numeric(Epiweek)), sep = "-"))

# Function to calculate start date 16 weeks before a given date
start_date <- function(date) {
  date - weeks(16)
}

# Define the end date
end_date <- ymd("2023-03-31")

# Unique lineages
lineages <- unique(air$Lineage)

# Loop through each lineage and create a scatter plot
for (lineage in lineages) {
  print(paste("Processing lineage:", lineage))  # Print the lineage being processed

  air_lineage <- air %>%
    filter(Lineage == lineage)

  # Get the first PHO clinical detection date for the current lineage
  first_pho_detection_date <- pho %>%
    filter(Lineage == lineage) %>%
    pull(Pho_Date)

  # Get the first GISAID clinical sample date for the current lineage
  first_gisaid_sample_date <- gisaid %>%
    filter(Lineage == lineage) %>%
    pull(Gisaid_Sample)
```

```
# Calculate the start date 16 weeks before the first clinical detection date
start_date_lineage <- start_date(first_pho_detection_date)

# Filter air_lineage based on the calculated start date and end date
air_lineage <- air_lineage %>%
  filter(Sample_Date >= start_date_lineage & Sample_Date <= end_date)

# Create breaks at weekly intervals
breaks <- seq(min(air_lineage$Sample_Date), max(air_lineage$Sample_Date), by = "week")

# Create labels for the breaks
labels <- paste(year(breaks), sprintf("%02d", as.numeric(as.week(breaks)$week)), sep = "-")

# Adjust breaks and labels for specific lineages
if (lineage %in% c("BQ*", "BA.2.75*", "BF*")) {
  breaks <- breaks[seq(1, length(breaks), by = 2)]
  labels <- labels[seq(1, length(labels), by = 2)]
}

# Define the order of legend labels
legend_order <- c("Airport Terminal 1 and 3", "Pooled Aircraft Sewage", "Peel", "Toronto", "York")

p <- ggplot(air_lineage,
        aes(x = Sample_Date, y = Frequency, color = Group)) +
  geom_point() +
  labs(title = paste("Emergence of ", lineage, "Lineages in Airport and surrounding municipal sites"),
     x = "Sample Date / Epi Week",
     y = paste("Frequency of ", lineage, "in Sample"),
     color = "Location") +
  scale_color_manual(values = c("Toronto" = "blue", "Peel" = "purple", "York" = "green",
                   "Pooled Aircraft Sewage" = "orange", "Airport Terminal 1 and 3" = "yellow2"),
           limits = legend_order) +
  scale_x_date(breaks = breaks, labels = labels) +
  geom_vline(xintercept = first_pho_detection_date, linetype = "dashed", color = "red") +
  annotate("text", x = first_pho_detection_date, y = Inf, label = paste("First PHO clinical detection of",
lineage),
       angle = 90, vjust = -0.5, hjust = 1, color = "red", size = 3) +
  geom_vline(xintercept = first_gisaid_sample_date, linetype = "dashed", color = "pink") +
  annotate("text", x = first_gisaid_sample_date, y = Inf, label = paste("First GISAID sample date of",
lineage),
       angle = 90, vjust = -0.5, hjust = 1, color = "pink", size = 3) +

  theme(axis.text.x = element_text(angle = 45, hjust = 1)) +
  scale_y_continuous(breaks = seq(0, max(air$Frequency, na.rm = TRUE), by = 0.1))  # Adjust y-axis
breaks

# Display plot
print(p)
```

```
  # Save plot
  ggsave(filename = paste0("scatter_plot_", lineage, ".png"), plot = p, width = 10, height = 6)
}
```

S:R346T Plot – R

```
suppressPackageStartupMessages({
  library(readxl)
  library(dplyr)
  library(magrittr)
  library(tidyr)
  library(ggplot2)
  theme_set(theme_bw())
  library(lubridate)
  library(epitools)
})

airS1 <- read.csv("all_samples_SR346T_mutations.csv")
airS <- airS1[, 1:4]
names(airS) <- c("Sample_Name", "Sample_Date", "Breadth_of_Coverage",
        "S:R346T")

airS <- airS %>%
  mutate(Location = gsub("(20\\d+)|(-\\d+)", "", Sample_Name)) %>%
  mutate(Location = ifelse(
    Location %in% c("Ashbridges", "AshbridgesBay", "AshbridgeBay"),
    yes = "Ashbridges",
    no = ifelse(
      Location %in% c("HighlandCreek", "Highland", "HighlandCR"),
      yes = "HighlandCreek",
      no = Location)
  )) %>%
  mutate(Group = case_when(
    Location %in% c("Humber", "Ashbridges", "HighlandCreek", "NorthToronto") ~ "Toronto",
    Location %in% c("As", "At") ~ "Pooled Aircraft Sewage",
    Location %in% c("A1", "A3") ~ "Airport Terminal 1 and 3",
    Location %in% c("P1", "P2") ~ "Peel",
    Location %in% c("Y1", "Y5", "Y6") ~ "York",
    TRUE ~ "Devan Missed One"
  ))
airS <- select(airS, -Sample_Name) %>%
  pivot_longer(cols = !c(Group, Location, Sample_Date, Breadth_of_Coverage),
        values_to = "Frequency", names_to = "Mutation") %>%
  mutate(Sample_Date = ymd(Sample_Date),
      Frequency = ifelse(Frequency > 1, 1, Frequency))
write.csv(airS, file = "summary_all_samples_SR346T_clean.csv")
```

```
rm(airS1)

airS <- read.csv("summary_all_samples_clean.csv") %>%
  # Remove samples with less than 20% BOC and no coverage of S:R346T (Frequency = -1)
  filter(Breadth_of_Coverage >= 20 & Frequency >= 0)

# Ensure Sample_Date is a Date object and add Epiweek and Year columns
airS <- airS %>%
  mutate(Sample_Date = as.Date(Sample_Date),
         Epiweek = as.numeric(as.week(Sample_Date)$week),
         Year = year(Sample_Date),
         Epiweek_Year = paste(Year, sprintf("%02d", as.numeric(Epiweek)), sep = "-"))

# Define the start and end dates
start_date <- ymd("2022-04-01")
end_date <- ymd("2023-03-31")

# Filter airS based on the calculated start date and end date
airS <- airS %>%
  filter(Sample_Date >= start_date & Sample_Date <= end_date)

# Create breaks at weekly intervals
breaks <- seq(min(airS$Sample_Date), max(airS$Sample_Date), by = "week")

# Create labels for the breaks
labels <- paste(year(breaks), sprintf("%02d", as.numeric(as.week(breaks)$week)), sep = "-")

# Adjust breaks and labels for readability
breaks <- breaks[seq(1, length(breaks), by = 2)]
labels <- labels[seq(1, length(labels), by = 2)]


# Define the order of legend labels
legend_order <- c("Airport Terminal 1 and 3", "Pooled Aircraft Sewage", "Peel", "Toronto", "York")

p <- ggplot(airS,
            aes(x = Sample_Date, y = Frequency, color = Group)) +
  geom_point() +
  labs(title = paste("Emergence of S:R346T mutation in Airport and surrounding municipal sites"),
       x = "Sample Date / Epi Week",
       y = paste("Frequency of S:R346T in Sample"),
       color = "Location") +
  scale_color_manual(values = c("Toronto" = "blue", "Peel" = "purple", "York" = "green",
                                "Pooled Aircraft Sewage" = "orange", "Airport Terminal 1 and 3" = "yellow2"),
                     limits = legend_order) +
  scale_x_date(breaks = breaks, labels = labels) +
  theme(axis.text.x = element_text(angle = 45, hjust = 1)) +
```

```
  scale_y_continuous(breaks = seq(0, max(airS$Frequency, na.rm = TRUE), by = 0.1))  # Adjust y-axis
breaks

# Display plot
print(p)

# Save plot
ggsave(filename = paste0("scatter_plot_SR346T.png"), plot = p, width = 10, height = 6)
```
